# Identification of Genes and Networks Driving Cardiovascular and Metabolic Phenotypes in a Mouse F2 Intercross

**DOI:** 10.1371/journal.pone.0014319

**Published:** 2010-12-14

**Authors:** Jonathan M. J. Derry, Hua Zhong, Cliona Molony, Doug MacNeil, Debraj Guhathakurta, Bin Zhang, John Mudgett, Kersten Small, Lahcen El Fertak, Alain Guimond, Mohammed Selloum, Wenqing Zhao, Marie France Champy, Laurent Monassier, Tom Vogt, Doris Cully, Andrew Kasarskis, Eric E. Schadt

**Affiliations:** 1 Rosetta Inpharmatics LLC, A wholly owned subsidiary of Merck & Co, Seattle, Washington, United States of America; 2 Basic Research, Merck & Co., Inc., Rahway, New Jersey, United States of America; 3 Institut Clinique de la Souris, Illkirch, France; Stanford University, United States of America

## Abstract

To identify the genes and pathways that underlie cardiovascular and metabolic phenotypes we performed an integrated analysis of a mouse *C57BL/6J x A/J* F_2_ (B6AF2) cross by relating genome-wide gene expression data from adipose, kidney, and liver tissues to physiological endpoints measured in the population. We have identified a large number of trait QTLs including loci driving variation in cardiac function on chromosomes 2 and 6 and a hotspot for adiposity, energy metabolism, and glucose traits on chromosome 8. Integration of adipose gene expression data identified a core set of genes that drive the chromosome 8 adiposity QTL. This chromosome 8 *trans* eQTL signature contains genes associated with mitochondrial function and oxidative phosphorylation and maps to a subnetwork with conserved function in humans that was previously implicated in human obesity. In addition, human eSNPs corresponding to orthologous genes from the signature show enrichment for association to type II diabetes in the DIAGRAM cohort, supporting the idea that the chromosome 8 locus perturbs a molecular network that in humans senses variations in DNA and in turn affects metabolic disease risk. We functionally validate predictions from this approach by demonstrating metabolic phenotypes in knockout mice for three genes from the *trans* eQTL signature, *Akr1b8, Emr1,* and *Rgs2*. In addition we show that the transcriptional signatures for knockout of two of these genes, *Akr1b8* and *Rgs2*, map to the F2 network modules associated with the chromosome 8 *trans* eQTL signature and that these modules are in turn very significantly correlated with adiposity in the F2 population. Overall this study demonstrates how integrating gene expression data with QTL analysis in a network-based framework can aid in the elucidation of the molecular drivers of disease that can be translated from mice to humans.

## Introduction

Classical genetic approaches to the study of complex phenotypes have historically been based on relating DNA variation to trait differences in populations from specific paired matings. These quantitative trait locus (QTL) mapping techniques have been successful in identifying regions of the genome that control phenotypic variation, but have been less productive when it comes to the identification of causative functional DNA variants or, more importantly, how these variants act at the molecular level to drive phenotypes [1]. More recently, a number of groups have shown how integration of intermediate molecular phenotypes, such as gene and protein expression levels, can be used to aid the reconstruction of these pathways and genes [2]–[6].

Obesity is a significant health burden in the developed world as a consequence of the associated co-morbidities of diabetes, cardiovascular disease, and hypertension [7]–[9]. Historically, rodents have been used as models of human obesity and hypertension because the genetic backgrounds and environmental influences can be controlled and because there is evidence that homologous genes are involved [10]–[12]. Multiple studies of adiposity and hypertension in genetic crosses from rats and mice have identified a large number of QTL associated with these traits [13]–[18].

Here we report results from a mouse F_2_ intercross population in which metabolic parameters, blood pressure, and echocardiography traits were measured and integrated with gene expression data from adipose, kidney, and liver. In addition to identifying a large number of clinical trait QTL we identified a locus on mouse chromosome 8 that is responsible for driving the expression of a large number of genes specifically in the adipose. Using an integrated approach, including network modeling, we predicted that this gene signature is causally associated with adiposity phenotypes. We present data to support this conclusion by showing metabolic phenotypes in three knockout mouse strains corresponding to genes from the signature. We also show that adipose signatures associated with these knockouts map to the predicted co-expression modules linked to adiposity in the F2 population.

## Results

### Cardiovascular and Metabolic Traits in F_2_ progeny of a *C57BL/6J x A/J* cross

An F_2_ population was derived from a *C57BL/6J* x *A/J* cross (B6AF2) and traits were measured in 360 male and female progeny using a phenotyping platform outlined in Figure S1. Mice were placed on a high-fat high-salt balanced diet at week 7 and maintained on this chow until termination at week 16. Five principle phenotyping components were used: blood pressure and heart rate by tail cuff at week 10; echocardiography at week 10; energy utilization by Oxymax at week 12; oral glucose tolerance test (OGTT) at week 13; intra-peritoneal insulin sensitivity test (IPIST) at week 14; and body composition by Dexascan at week 15. In addition, a number of endpoints relevant to size and adiposity, and serum for blood analytes including lipids, were collected at final necropsy. Table S1 shows a list of traits and the mean +/−SD values in the parental, F_1_, and F_2_ populations.

### Mapping of QTL for body composition, echocardiography, blood pressure, and cholesterol traits

A genetic map was derived from the genotype data for the F_2_ progeny and used to identify trait QTL. Table 1 shows the 51 genome-wide significant trait QTL (LOD≥4.3/FDR = 0.10) [19] that were mapped for a total of 27 selected traits. A full set of QTL for all traits calculated for all animals, as well as males and females separately, is given in Table S2.

**Table 1:** Significant trait QTL mapped in the B6AF2 cohort.

Trait classification	Trait Name	Sub-trait Name	Time-point	Chr	QTL Peak (cM)	LOD	Ref
Adiposity	Mesenteric Fat	% Mesenteric Fat	W16	6	35	4.6	[21]
Adiposity	Mesenteric Fat	% Mesenteric Fat	W16	8	34	6.7	
Adiposity	Mesenteric Fat	% Mesenteric Fat	W16	16	2	4.3	
Adiposity	Subcutaneous Fat	% Subcutaneous Fat	W16	2	26	4.4	
Adiposity	Subcutaneous Fat	% Subcutaneous Fat	W16	8	34	6.1	
Adiposity	Total Fat	% Total Fat (DEXA)	W15	2	21	4.8	
Adiposity	Total Fat	% Total Fat (DEXA)	W15	6	34	5.6	[21]
Adiposity	Total Fat	% Total Fat (DEXA)	W15	8	43	8.9	
Adiposity	Gonadal Fat	Absolute Gonadal Fat	W16	6	33	4.6	[21]
Adiposity	Gonadal Fat	Absolute Gonadal Fat	W16	8	34	10.9	
Adiposity	Mesenteric Fat	Absolute Mesenteric Fat	W16	6	36	6.7	[21]
Adiposity	Mesenteric Fat	Absolute Mesenteric Fat	W16	8	34	6.4	
Adiposity	Mesenteric Fat	Absolute Mesenteric Fat	W16	16	2	6	
Adiposity	Subcutaneous Fat	Absolute Subcutaneous Fat	W16	6	33	5.9	[21]
Adiposity	Subcutaneous Fat	Absolute Subcutaneous Fat	W16	8	34	6.3	
Adiposity	Total Fat	Absolute Total Fat (DEXA)	W15	6	34	6.6	[21]
Adiposity	Total Fat	Absolute Total Fat (DEXA)	W15	8	43	5.6	
Blood Analytes	Potassium	Potassium	W16	9	0	5.1	
Blood Pressure	Heart Rate	Heart Rate	W10	1	49	4.5	
Blood Pressure	SBP	Systolic Blood Pressure	W10	1	56	4.8	[10], [18]
Bone	BMD	Bone Mineral Density	W15	10	60	5	
Bone	BMD	Bone Mineral Density	W15	14	13	4.3	
Echocardiographic	Aorta Diameter	Aorta Diameter	W10	6	42	5.3	
Echocardiographic	Velocity Time Integral	Velocity Time Integral	W10	2	53	5.4	
Echocardiographic	Ejection Fraction	Ejection Fraction	W10	17	13	4.5	
Glucose/Insulin	OGTT	OGTT AUC	W13	6	47	5.6	[65]
Glucose/Insulin	OGTT	OGTT Glucose Change (0–15 min)	W13	8	31	6.1	
Leanness	Lean mass	% Lean Mass (DEXA)	W15	2	21	4.5	
Leanness	Lean mass	% Lean Mass (DEXA)	W15	6	33	5.8	[21]
Leanness	Lean mass	% Lean Mass (DEXA)	W15	8	43	8.7	
Lipids	HDL	HDL Cholesterol	W16	4	49	5	[66]
Lipids	LDL	LDL cholesterol	W16	6	52	6.3	
Lipids	Triglyceride	Triglyceride	W16	8	36	4.9	
Size	Heart	Heart weight/Body weight	W16	6	30	7.7	
Size	Heart	Heart weight/Body weight	W16	8	49	8.1	
Size	Heart	Heart-Left ventricle weight/BWt	W16	8	34	6.8	
Size	Kidney	Kidney weight/Body weight	W16	8	34	16.1	
Size	Kidney	Kidney weight/Body weight	W16	16	6	4.6	
Size	Liver	Liver weight/Body weight	W16	8	32	13.8	
Size	weight	Weight	W6	1	64	4.8	
Size	weight	Weight	W7	6	31	5.1	[21]
Size	weight	Weight	W8	1	64	4.4	[20]
Size	weight	Weight	W10	1	71	4.5	
Size	weight	Weight	W10	6	31	4.7	[21]
Size	weight	Weight	W12	1	64	5.1	
Size	weight	Weight Change W6-W16	W16	1	78	4.5	
Size	weight	Weight Change W6-W16	W16	6	47	5.8	[21]
Size	weight	Weight Change W6-W16	W16	8	43	4.4	
Size	weight	Weight Change W6-W16	W16	16	2	5.9	
Size	weight	Weight Change W12-W16	W16	1	64	6.2	
Size	weight	Weight Change W12-W16	W16	6	42	4.9	[21]

QTL Peak is the map position corresponding to the maximum LOD score.

Notable hotspots for QTL associated with body composition, size, and glucose/insulin traits mapped to chromosomes 1, 6, and 8. These QTL likely correspond to QTL for weight previously reported by Zhang and Gershenfeld in intercross (F2) and backcross (N2) populations from these strains [20]. These authors reported a significant QTL for weight at week 8, *Bw8q1* on chromosome 1, as well as a suggestive QTL on chromosome 8 for the same trait, and a suggestive QTL for 2-week weight gain on chromosome 6 [20]. In addition, Shao et al have used chromosome substitution strains to dissect the chromosome 6 QTL and provide evidence that it is a complex QTL consisting of four distinct loci [21].

**Figure 1:**
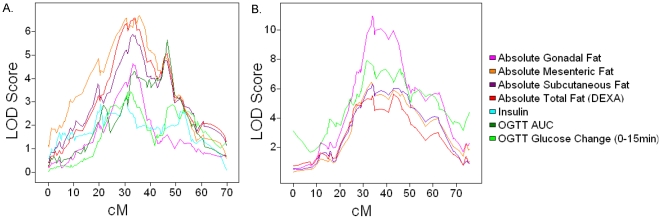
QTL hotspots for body composition, insulin, and glucose traits on chromosomes 6 and 8. QTL plots for traits mapping to chromosome 6 (A) and chromosome 8 (B) indicate that shared DNA variation likely drives multiple metabolic phenotypes on the respective chromosomes. For clarity not all significant QTL mapping to these loci are shown (see Table 1).

QTL hotspots associated with composition and glucose/insulin traits on chromosomes 6 and 8 in this study are shown in Figure 1. Both of these loci have effects on multiple fat depots in the mouse including gonadal, mesenteric, and subcutaneous adipose. In addition, % lean mass and tissue weight traits, as a fraction of total weight of the mouse, also map to these loci. This probably reflects secondary effects of the increases in fat depots rather than a direct effect on these traits. This hypothesis is supported by the observation that absolute measures of either lean mass or tissue weight fail to show significant or suggestive QTL at these loci. We conclude that the main effects of the chromosome 6 and chromosome 8 loci with regard to body composition are on adiposity. In addition, traits related to maintenance of blood glucose levels map to both loci while traits relevant to energy metabolism (Heat, VO2, and VC02) and triglyceride level are present on chromosome 8 (Figure 1, Figure S2, Table 1, and Table S2). Overall, these two loci regulate traits that in humans are associated with metabolic syndrome (obesity, diabetes, and lipid-related traits). While the adipose traits show very significant correlation across the population, the lipid and glucose/insulin-related traits show distinct distributions across individuals suggestive of complex genetic control (Figure S3). A subsequent section investigates the genes and networks that are driving this phenotypic variation (see below).

**Figure 2:**
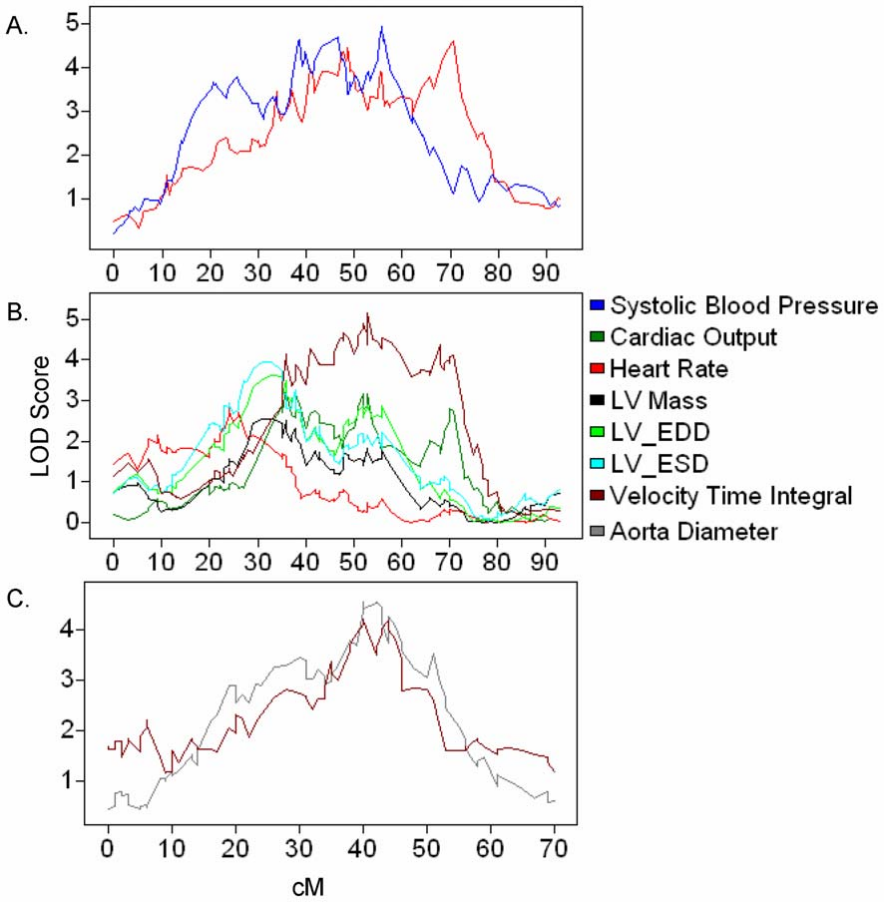
QTLs associated with blood pressure, heart rate, and echocardiography traits highlight different aspects of cardiovascular physiology. QTL plots showing LOD scores by chromosomal position for the indicated traits. (A) locus on chromosome 1 associated with variation in blood pressure and heart rate; (B) locus on chromosome 2 associated with left ventricular anatomy and function and its relationship to cardiac output; (C) locus on chromosome 6 regulating cardiac output parameters associated with aorta diameter.

We detected a number of QTL associated with blood pressure, heart rate, and echocardiography traits (Figure 2). On chromosome 1 we identified an overlapping QTL for systolic blood pressure and heart rate (QTL peak  = 56 cm & 49 cM; maximum LOD = 4.8 & 4.5, respectively) (Figure 2A). This probably corresponds to the *Abbp1* locus identified by Woo et al in a similar strain background [18]. These investigators identified additional QTL: *Abbp2* on chromosome 4 at 25 cm, *Abbp3* on chromosome 7 at 25 cM, and *Abbp4* on chromosome 11 at 58 cM. Interestingly, we detected no signal above LOD = 3 for a blood pressure QTL on chromosome 11 but did detect a QTL on chromosome 7 (QTL peak = 47 cM; maximum LOD = 4.2) and a QTL on chromosome 4 (QTL peak = 19 cM; maximum LOD = 3.0) when we adjusted for serum insulin levels (Table S2). In regard to electrocardiographic traits, we identified a cluster on mouse chromosome 2 containing QTL for velocity time integral, cardiac output, heart rate, LV mass, LV_EDD, and LV_ESD (Figure 2B). These traits relate to cardiac structure and function, in particular left ventricular function, indicating the presence of a locus on chromosome 2 that regulates overall cardiac output. In this regard, it is interesting to note that Suzuki et al have mapped a genetic modifier of murine dilated myopathy (*Hrtfm1*) to an overlapping region of chromosome 2 in a *C57BL/6J x DBA/2J* backcross [22]. In comparison with the chromosome 2 locus that appears to drive variation in cardiac output related to left ventricular function, we also identified a locus on chromosome 6 in which velocity time integral associates with aorta diameter (Figure 2C).

Significant QTL for serum lipid levels were identified on chromosome 4 (HDL cholesterol; QTL peak = 49 cM; maximum LOD = 5.0), chromosome 6 (LDL cholesterol; QTL peak = 52 cM, maximum LOD = 6.3), and chromosome 8 (triglyceride; QTL peak = 5 cM, maximum LOD = 4.9). In addition to the trait QTL identified above, additional loci were mapped for the broad trait areas of blood analytes/potassium on chromosome 9 (QTL peak = 0 cM; maximum LOD = 5.1), and bone on chromosomes 10 and 14 (BMD; QTL peak = 60 cm and 13 cm, respectively; maximum LOD = 5.0 and 4.3, respectively). It is noteworthy that we did not detect genome-wide significant QTL (LOD≥4.3/FDR = 0.10) for all of the traits measured in the B6AF2 cohort. In particular, we did not detect significant QTL for any of the IPIST or Oxymax measurements or for serum measures of glucose, insulin, creatinine, chloride, sodium, and urea in analyses that included both genders, although suggestive QTL (LOD>3.0) were identified in these analyses and significant QTL were identified in the gender-specific analyses (Table S2).

### Mapping of eQTL in liver, adipose, kidney medulla, and kidney cortex

Whole genome expression data were generated for all F_2_ progeny for four different tissues (gonadal adipose, liver, kidney cortex, and kidney medulla) using a custom Agilent mouse array containing ∼40,000 unique reporter sequences. Each reporter sequence was individually considered as a trait for each tissue and used to map expression QTL (eQTL). The distribution of LOD scores by tissue is shown in Figure S4A. eQTL were considered as *cis* acting QTL if the QTL mapping position lay within +/−20 Mb of the physical gene location. The bin size for consideration of *cis* acting eQTL was similar to those used in previous studies and was derived from the data in Figure S4B, which show a significant increase in the number of eQTL detected within 20 Mb of the associated gene only when the gene and QTL reside on the same chromosome. A list of all eQTL with LOD≥5, corresponding to an FDR<0.05 in all tissues, is given in Table S3 (all animals), Table S4 (males only), and Table S5 (females only). We also assessed the degree of sharing of eQTLs between tissues (Table S6). In order to do this we defined eQTL at a reporter, chromosome, tissue level (that is an eQTL was counted once as the maximum LOD > = 5 for any particular reporter, chromosome, tissue group). Pairwise comparison of tissues shows greatest sharing of eQTLs for the kidneycortex:kidneymedulla comparison (38.4% cis, 11.9% trans). Overall there is much greater consistency of *cis* eQTLs between tissues (average = 27.6%) than *trans* eQTLs (average = 8.9%), consistent with previous observations.

### An adipose trans eQTL signature drives the body composition QTL on chromosome 8 in the B6AF2 population

Inspection of the eQTL distribution across chromosomes for different tissues identified an eQTL hotspot in adipose between 30 and 40 cM on chromosome 8, as shown in Figure S5 (expected number of eQTL in 10 cM interval = 62; actual number  = 1722; *P* = 2.5e^−321^). While trans eQTL hotspots can result from artifacts we do not believe this is the case for this signature for three reasons. First, when we permute the eQTL data and plot the eQTL numbers per 10 Mb genomic bin across the genome we never see more than 38 eQTL per bin, compared with >1000 in the observed data for the chromosome 8 region (Figure S6). Second, if it was the result of population substructure in the intercross population we would anticipate seeing an enrichment of eQTL at this locus across tissues while we actually observe that the hotspot is specific to adipose (Figure S5). Most importantly, we have been able to replicate this finding in an independent B6AF2 cohort of comparable size (“Jaxshort BxA”). Specifically we find that of the 1,565 eQTLs (LOD> = 5) in the MCI BxA signature at the chromosome 8 locus that are *trans* (gene and QTL on different chromosome), 1,491 (95%) replicate on chromosome 8 in the Jaxshort BxA cohort at a LOD> = 2. With more stringent replication criteria, that requires LOD> = 3 and maximum QTL LOD position in the 30–40 cM interval, 929 (59%) eQTL replicate. Comparison of the LOD scores for the eQTLs mapping to chromosome 8 in adipose in the two crosses shows significant correlation (R2 = 0.25, p = 7.8e-96; Figure S7) and comparable location (Figure S8). In the Jaxshort BxA cross the hotspot is specific to adipose, as it is in MCI BxA; in this case specificity is compared to liver, muscle and hypothalamus. This replication data for the Jaxshort BxA cross is included as a series of tables containing the gene expression data for the chromosome 8 eQTL genes (Table S7), the genotypes for all animals (Table S8), the genders of individuals (Table S9). Together all these data provide convincing evidence that the trans eQTL hotspot is unlikely to be an artifact.

More than 95% of the eQTL at this locus are *trans* eQTL, therefore we refer to this as the "*trans8_eQTL"* signature. This location coincides with the position of the body composition QTL hotspot (Figure 1B). To explore whether the trans8_eQTL signature influences these traits we looked at the relationship between the expression of genes in the signature in adipose tissue and various endpoints. The first principal component of the trans8_eQTL signature was computed (ch8PC1) and correlated to the trait values across the population. The correlation coefficients and associated *P* values are shown in Table 2. These data indicate significant correlation of the trans8_eQTL signature with a number of the traits at this locus. This correlation structure could be the result of gene expression traits driving the clinical traits in a causal relationship, or alternatively the gene expression changes could lie downstream of the variation in the clinical traits. To distinguish these possibilities we specifically looked for enrichment of gene expression traits that tested as causal for the individual clinical traits at this locus using a previously described causality test [5]. We calculated the fold enrichment for genes testing causal for a trait at this locus compared with across the genome by computing the number of reporter_ids from the chromosome 8 eQTL signature testing causal for trait/total reporter_ids in the chromosome 8 eQTL signature as a ratio to the total number of reporter_ids testing causal for the trait/total number of reporter_ids on the array and used Fisher's exact test to estimate the significance of these enrichments. In this way we identified enrichments for causal genes linked to metabolic traits in the trans8_eQTL signature of up to 18.6-fold with highly significant associated *p* values (Table 2 and Table S10). For example, 75% of the reporters on the array chip that are expressed in adipose and test causal for % mesenteric fat are present in the trans8_eQTL signature (243 reporters out of 322 genome-wide total testing causal for this trait: Table S10).

**Table 2:** Association of eQTL signature to trait QTL on chromosome 8.

Sub-trait Name	Correlation to PC	Correlation *p* value	Fold enrichment for causal genes	Enrichment *p* value
% Total Fat (DEXA)	0.61	1.4E-31	7.5	7.7E-24
Absolute Gonadal Fat	0.58	8.5E-28	13.3	2.7E-42
Absolute Total Fat (DEXA)	0.54	8.2E-24	12.4	8.7E-144
% Mesenteric Fat	0.50	3.7E-19	18.6	3.3E-176
% Subcutaneous Fat	0.50	1.6E-18	16.5	2.6E-154
Absolute Mesenteric Fat	0.48	1.7E-18	9.7	2.5E-147
Absolute Subcutaneous Fat	0.47	7.4E-18	8.6	1.5E-95
Weight Change W6-W9	0.42	9.0E-14	14.6	7.3E-41
Triglyceride	0.34	3.0E-09	14.0	3.1E-273
Heart weight/Body weight	−0.53	1.0E-22	1.4	3.0E-01
OGTT Glucose Change (0–15 min)	0.24	3.4E-05	12.0	2.9E-11
% Lean Mass (DEXA)	−0.62	3.4E-32	7.2	1.1E-20

Fold enrichment obtained by calculating: (# reporter_ids from ch8 eQTL signature testing causal for trait/total reporter_ids in ch8 eQTL signature)/(total # reporter_ids testing causal for trait/total reporter_ids on array).

Enrichment p value is from Fisher's exact test.

PC = 1st principal component for chromosome 8 eQTL signature.

Annotation of the trans8_eQTL signature by enrichment analysis relative to genes in the Gene Ontology (GO) catalogue indicates that these genes are strongly associated with cellular metabolic processes, particularly oxidative phosphorylation and mitochondrial function (Figure S9). The idea that the genes in this signature relate to mitochondrial function is further supported by the observation that 307 out of 1537 signature genes are also found in a set of 1098 genes whose protein products have been shown localize to mitochondria (enrichment *p* value = 3.36e^−144^) [23]. We also mapped the signature to gene co-expression networks. Gene co-expression network analysis (GCENA) has been used to identify gene subnetworks and to prioritize gene targets associated with a variety of common human diseases such as cancer and obesity [24]–[28]. One important end product of GCENA is the construction of gene modules comprised of highly interconnected genes, and a number of studies have demonstrated that co-expression network modules are generally enriched for known biological pathways, for genes that are linked to common genetic loci, and for genes associated with disease [24]–[32]. The topological overlap map of the MCI BxA adipose is shown in Figure S10. Intersection of the genes from the trans8_eQTL signature indicates that they are very highly enriched in the red (9.4-fold enriched; *p* = 1.13E^−132^) and turquoise (2.2-fold enriched; *p* = 1.14E^−26^) modules of the co-expression network. Indeed ∼70% of the genes in the red module are regulated by the chromosome 8 locus indicating this is a key genetic driver of this subnetwork. Annotation of these modules shows that the red network is negatively correlated with adiposity traits and is enriched in GO terms associated with a variety of catabolic and metabolic processes and with mitochondrial function. The turquoise module is positively correlated with adiposity traits and is linked to a diverse set of biological processes including angiogenesis, apoptosis, cell cycle, and immune cell activation (Figure S10).

We sought also to place this signature in the context of datasets relevant to human obesity. In this context, the trans8_eQTL signature shows strong enrichment in a human adipose co-expression network module that we previously demonstrated to be associated with BMI in humans (Figure S11) [25]. Specifically, genes in the trans8_eQTL signature map to two expression modules in the human adipose connectivity map. The red module consists of genes involved in mitochondrial function while the turquoise module is enriched for genes associated with immune response. Both modules show correlation with metabolic traits (leptin, BFM, BMI, HDL cholesterol, waist, and weight) and the turquoise module has been identified as a key driver of obesity traits in humans [25]. Together, these data support a role for the chromosome 8 locus in driving adiposity phenotypes via effects on energy metabolism and through genes and networks that are conserved in mouse and human.

### Validation of effects of chromosome 8 trans eQTL signature genes on obesity traits

We selected three genes from the trans8_eQTL signature for validation experiments in knockout mice. These genes were selected based on a number of criteria, including causality for adiposity traits based on the *trans* eQTL in the B6AF2 cohort, causality for adiposity traits across other F_2_ crosses [5], [6], [33], and availability of genetically modified mice. It is noteworthy that *Akr1b8*, *Emr1*, and *Rgs2* are in the top 5% of genes testing causal for obesity traits in our database.

**Figure 3:**
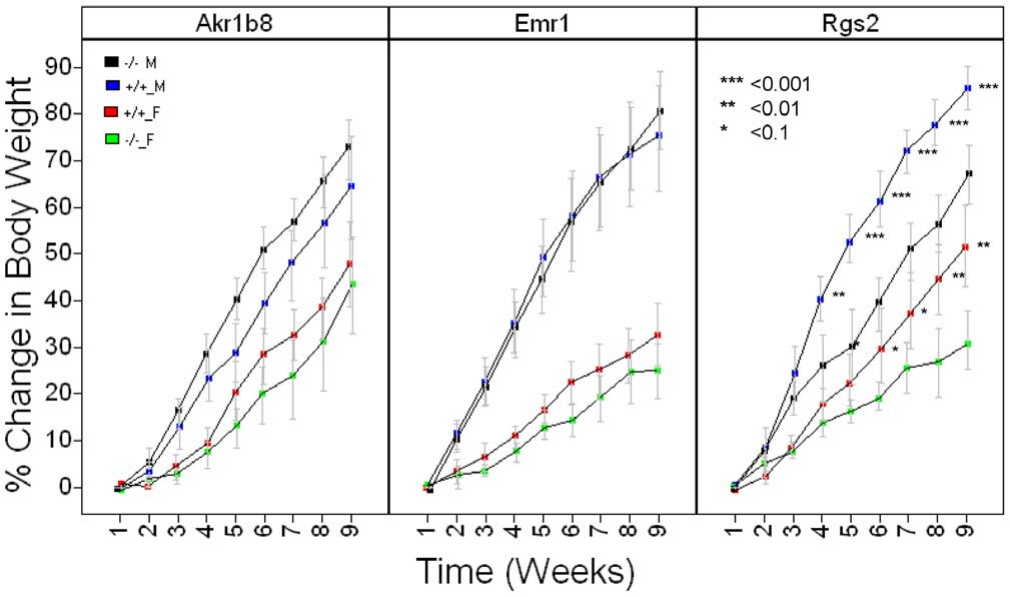
Growth Curves for *Akr1b8^−/−^, Emr1^−/−^* and *Rgs2^−/−^* mice. *Akr1b8, Emr1* and *Rgs2* knockout (n = 9 per gender)* and littermate control (n = 9 per gender)^#^ mice were placed on HFD at 9, 11, and 9 weeks of age respectively (W1 above). Body weights were recorded weekly for a total of 9 weeks. Statistically significant differences between genotypic groups split by gender are marked. Blue  = WT males; Black  = KO males; Red = WT females; Green  =  KO females. *actual number of *Emr1* ^+/+^ female mice  = 8.^ #^actual number of *Rgs2^−/−^* female mice = 6.

We first looked at the response to dietary challenge by maintaining knockout mice and wild-type littermate controls on a high-fat diet (HFD) for 9 weeks and recording changes in body weight. The *Rgs2* line showed statistically significant genotypic differences in body weight gain in response to a HFD (Figure 3 and Table S11). This is consistent with the causality prediction of an effect of *Rgs2* on body weight (Table 3). Both male and female *Rgs2* knockout mice gained less weight than littermate controls after 9 weeks of HFD (67% v 86% for males, *p* = 2.8e^−4^; 32% v 52% for females, *p* = 5.7e^−3^). These effects do not appear to be the consequence of lower food intake, suggesting a mechanism linked to energy metabolism rather than to hypophagia (data not shown).

**Table 3:** Summary of Causality and Knockout Phenotype Data for *Akr1b8, Emr1*, and *Rgs2*.

			Knockout Phenotypes		
Gene Symbol	Traits for which genes test causal based on eQTL/cQTL overlap	Direction of correlation of expression to trait	Change Body Weight on HFD	Body Composition on chow or HFD	Serum Lipid
*Akr1b8*	% gonadal fat	negative	No difference KO v WT for males or females	Increased % Fat in KOs on chow or HFD compared to WTs (males)	Increased serum cholesterol in KOs on HFD compared to WTs (males)
*Emr1*	Weight, Absolute Subcutaneous adipose, Absolute gonadal adipose, Absolute mesenteric adipose, weight change W6-W12	positive	No difference KO v WT for males or females	Decreased % Fat in KOs on chow or HFD compared to WT (females)	No difference in serum lipids KO v WT for males or females
*Rgs2*	Weight, Absolute Total Fat, % Total Fat, % subcutaneous adipose, Absolute mesenteric adipose, absolute gonadal adipose	positive	Male and female KO mice gain less weight on HFD	Decreased % Fat in KOs in response to HFD compared to WT (males)	Increased serum cholesterol in KOs on chow compared to WTs
*Rgs2*	% Lean Mass (DEXA)	negative	Male and female KO mice gain less weight on HFD	Decreased % Fat in KOs in response to HFD compared to WT (males)	Increased serum cholesterol in KOs on chow compared to WTs

**Figure 4:**
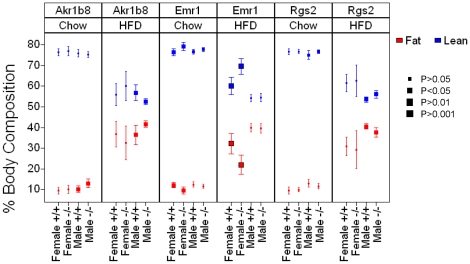
Body Composition by qNMR for *Akr1b8^−/−^, Emr1^−/−^* and *Rgs2^−/−^* mice on chow and HFD. Body composition for *Akr1b8, Emr1* and *Rgs2* knockout and littermate control mice was assessed on chow diet (8–10 weeks of age) and after 9 weeks on HFD (17–23 weeks of age). Data points are averages with 95% confidence intervals. Statistically significant differences between genotypic groups split by gender are marked. All gender, genotype, diet groups were n = 9 except for the female *Emr1^+/+^* HFD fed and female *Rgs2^−/−^* chow fed groups were n = 8.

We also compared the body composition of knockout and wild-type mice measured by qNMR at two time-points, before and after HFD feeding (Figure 4 and Table S12). We observed that the *Rgs2^−/−^* mice gained less overall weight on HFD and that the male knockout mice were leaner than the wild-type mice (37.6+/−2.1% fat versus 40.5+/−1.2% fat; *p* = 2.6e^−3^). The female *Rgs2* mice did not show a statistically significant difference between the genotypic groups in body composition after HFD. A similar sexual dimorphism towards a male effect was seen in the *Akr1b8^−/−^* strain, although in this case the tendency was for the male knockout mice to be fatter than their wild-type littermate controls on both chow (13.0+/−2.1% fat versus 10.1+/−1.6% fat; *p* = 4.4e^−3^) and HFD (41.7+/−1.5% fat versus 36.4+/−4.6% fat; *p* = 4.9e^−3^) (Figure 4 and Table S12). The male *Akr1b8^−/−^* mice also had higher serum cholesterol levels after HFD (7.50+/−0.58 mmol/L versus 6.33+/−0.76 mmol/L; *p* = 2.0e^−3^) reflecting increases in both HDL and LDL cholesterol (Table S13). The *Emr1^−/−^* strain showed larger effects in the female than the male mice, with the knockout showing a strong tendency to be leaner, particularly in response to a HFD (22.0+/−4.6% fat versus 32.2+/−5.0% fat; *p* = 5.2e^−4^). Overall, these data validate our predictions from genetic analysis that these genes are involved in driving adiposity traits in mice (as summarized in Table 3). Specifically, our genetic analysis indicated a causal relationship between *Akr1b8, Emr1,* and *Rgs2* expression level and measures of body adiposity, with a negative correlation between *Akr1b8* expression in adipose and % fat, and a positive correlation between *Emr1* and *Rgs2* expression and % fat. All of these predictions were recapitulated in knockout mice, in which loss of *Rgs2* led to increased % fat and loss of *Emr1* or *Rgs2* to decreased % fat. These data also underscore the strong influence of gender on metabolic traits in mice, an observation we have previously noted [34].

We also analyzed the transcriptional consequences of knocking out the *Akr1b8* and *Rgs2* genes; unfortunately tissues were not available from the *Emr1^−/−^* mice for profiling analysis. Specifically we sought to explore whether the adipose knock out signatures from these mice were enriched in the network modules corresponding to the trans8_eQTL signature, namely the red and turquoise modules that show strong correlation with adiposity traits (Figure S10). This analysis (Table S14) indicates that the gene expression signature in adipose associated with deletion of the *Akr1b8* gene is very significantly enriched specifically in the turquoise module (fold enrichment = 1.77-fold; hypergeometric *P* value = 0) but not in the red module (fold enrichment = 1.02-fold; hypergeometric *P* value  = 0.49). The *Rgs2* signature shows modest fold enrichments in both the turquoise (fold enrichment = 1.21-fold; hypergeometric *P* value = 0.004) and the red (fold enrichment = 1.45-fold; hypergeometric *P* value = 0.02) modules although it only reaches significance *P*<0.01 in the turquoise module. Overall this analysis supports the view that the *Akr1b8* and *Rgs2* genes are important regulators of adiposity in the mouse and that their perturbation leads not only to predicted metabolic changes in the mouse but also to changes in the transcriptional networks in which they operate.

### Trans8_eQTL signature is enriched for genes that show association to T2D in human GWAS

In addition to being linked to adiposity traits, the *trans* eQTL signature is associated with endpoints relevant to diabetes, namely glucose traits (Table 1, Table S2, and Figure S2). We reasoned that if this set of genes is important in human disease, SNPs linked to these genes would show enrichment of association with particular disease phenotypes. Toward this end, we identified human eSNPs (SNPs that significantly associate with expression traits) corresponding to the orthologous human genes from the trans8_eQTL signature using data from a genetics of gene expression (GGE) study in an obesity cohort comprised of 800 individuals from which liver, subcutaneous, and omental adipose tissues were collected (Zhong, H. et al. Elucidating Networks of eSNPs Associated with Type 2 Diabetes. Submitted). To test whether the eSNPs were enriched for association to type 2 diabetes (T2D), we assembled GWAS results from the meta-analysis of multiple T2D cohorts, referred to here as the DIAGRAM study [35].

Among the 1537 genes, 1249 have human orthologs, of which 502 have at least one eSNP in adipose tissue. The distribution of their T2D association *P* values from the DIAGRAM (referred to here as *P_T2D_*) is shown in Figure S12. It is apparent that the *P_T2D_* distribution of the eSNPs associated with genes in the trans8_eQTL signature is enriched for SNPs associated with T2D, in that the eSNP *P_T2D_* values are skewed towards the significance end of the *P_T2D_* spectrum. To statistically estimate the degree of enrichment and associated significance, we empirically estimated the null distribution by randomly sampling 100,000 sets of SNPs from the DIAGRAM data such that the SNP set size, the location distribution of the SNPs with respect to protein coding genes, the linkage disequilibrium structure, and the minor allele frequency matched that of the eSNP set (Methods S1 and Materials and Methods). We found that 6.85% of SNPs in the eSNP set (460 out of 6,720 SNPs) had *P_T2D_* values <0.05, compared with an average of 5.62% SNPs (95% CI: 5.01% to 6.17%) in the random sets (Z = 4.38; *P* = 6.0e^−06^). It is of note that the eSNP sets corresponding to the trans8_eQTL signature generated from subcutaneous and omental adipose tissue separately were both enriched for lower *P_T2D_* values: for the omental adipose 394 SNPs out of 5,639 SNPs (6.99%) had *P_T2D_* values <0.05; for the subcutaneous adipose 338 SNPs out of 4,841 SNPs (6.98%) had *P_T2D_* values <0.05 (data not shown). Therefore, this enrichment is consistently observed for the trans8_eQTL signature.

## Discussion

This study contributes significantly to our knowledge of QTL in mouse that genetically regulate traits relevant to metabolic and cardiovascular disease, as well as hypertension. Furthermore, the tissue gene expression data provided in this paper provide a powerful framework for relating DNA variation to gene expression changes, and in turn to phenotypic variation. We have shown how this can be applied to help elucidate the molecular mechanisms underlying complex trait variation in the context of a chromosome 8 adiposity QTL.

The emphasis of our approach is not to directly define the *cis* variants that underlie QTL but rather to understand how this variation drives changes in entire networks of genes that regulate physiological processes. For example, it is unclear from our analysis what the underlying perturbation(s) on chromosome 8 that drive the trans8_eQTL signature are, but it is apparent that the genes whose expression traits map here in *trans* are the molecular effectors of the *cis* signal. We have shown this by demonstrating: a) that the adipose expression of genes in the trans8_eQTL signature is highly correlated with the adiposity-related traits; b) that the genes test causal for driving variation in the adiposity traits; c) that the genes map to modules in human adipose that have been implicated in human obesity; and d) by validating three of the genes in the signature by phenotyping knockout mice.

Annotation of the trans8_eQTL signature by reference to existing datasets strongly suggests that the mechanism underlying the effects of the chromosome 8 DNA variation on adiposity relates to energy expenditure. The genes in the signature are highly enriched for metabolic processes associated with generation of precursor metabolites, oxidative phosphorylation, and mitochondrial ATP synthesis. Remarkably, 20% of the genes are found within a curated set of 1090 genes with protein products predicted to be located in the mitochondrion [23]. Given that the curated mitochondrial gene set is highly unlikely to be comprehensive, the true fraction of the genes in the trans8_eQTL signature that localize to the mitochondrion is likely to be much higher. In this context it is interesting to note that one of the *cis* acting candidates at the chromosome 8 locus is *Fto*, a gene that has been robustly validated for association with human obesity [36]–[38] and has recently been implicated in regulating energy expenditure in mice [39]. *Fto^−/−^* mice show significantly elevated oxygen consumption (V02), carbon dioxide production (VCO2), and calculated heat production (Heat) relative to their wild-type littermates. Consistent with an *Fto* driven mechanism underlying the chromosome 8 locus, there are highly suggestive QTL at this locus for traits associated with energy metabolism (Figure S2 and Table S2). *FTO* is also associated with T2D in human populations [40]–[42]. In this regard it is interesting that we were able to demonstrate that genes in the signature show greater association with T2D than similar sets of randomly selected genes (Figure S12). An alternative candidate gene on chromosome 8 that has a well-established role in adiposity and mitochondrial function is *Ucp1* [43]; however, we did not detect a significant *cis* eQTL for *Ucp1* in B6AF2 adipose, nor are there known non-synonymous SNPs in the gene between these two strains. Furthermore, we detected a significant adipose *cis* eQTL for *Ucp1* in a *C57BL/6J ob/ob* vs. *BTBR ob/ob* F2 cross [44] but this *cis* eQTL is not associated with the trans8_eQTL signature or a significant adiposity QTL on chromosome 8. We conclude that *Fto* is a good candidate for explaining the chromosome 8 adiposity QTL although additional studies into *Fto* perturbation in mice will be necessary to validate this connection.

We have shown experimentally the effects of deleting three genes regulated by the chromosome 8 locus on adiposity traits in knockout mice. These genes are *Emr1, Rgs2,* and *Akr1b8,* and the associations with metabolic phenotypes represent novel findings. *Emr1* is the prototypical macrophage lineage marker in the mouse, although its tissue distribution appears to be different in humans [45]. It is a seven-membrane spanning molecule (TM7) and, together with CD97, EMR2, EMR3, and EMR4, belongs to the EGF-TM7 family, a subfamily within the adhesion class of TM7 receptors [46]. Although these receptors have variable numbers of EGF-like domains that can mediate ligand binding, a cellular ligand for *Emr1* has not yet been identified. *Emr1*-deficient mice are healthy and do not show abnormalities in macrophage development and function [46]. *Rgs2* acts downstream of TM7 proteins to regulate and integrate G-protein coupled signaling [47]. Specifically, RGS2 is a selective and potent inhibitor of G_αq_ signaling [48], is ubiquitously expressed throughout the cardiovascular system, and is capable of inducing adipocyte differentiation [49]. *Rgs2*-null mice are hypertensive [50]–[52] and polymorphisms in RGS2 have been implicated in human hypertension [53], [54]. More recently, RGS2 variation has been associated with metabolic syndrome and weight gain in humans [55], [56]. *Akr1b8* is a member of the aldo-keto reductase (AKR) superfamily encompassing more than 140 proteins with different physiological roles, most of which are NAD(P)(H)-dependent oxidoreductases that metabolize carbohydrates, steroids, prostaglandins, and other endogenous aldehydes and ketones, as well as xenobiotic compounds. This family is thought to play physiological roles in osmotic homeostasis, steroid and xenobiotic metabolism, signal processing, and oxidative defense mechanisms [57]. All three of these genes show association with the trans8_eqtl signature and each displays a unique pattern of metabolic dysfunction when knocked out in mice.

In addition to the chromosome 8 eQTL hotspot in adipose there are other potential hotspots in other tissues. For example, there is an enrichment of eQTLs on chromosome 6 in liver, on chromosome 18 in liver and kidney cortex, on chromosome 1 in liver and adipose, and on chromosomes 4 and 13 in kidney cortex and kidney medulla (Figure S5). While all of these are significant with respect to the permuted eQTL data we have been unable to replicate them in an independent cohort in the same way as we have demonstrated for the chromosome 8 adipose eQTL hotspot; the chromosome 6 liver eQTL hotspot is present in the Jaxshort BxA cohort but is much weaker. Therefore at this point it appears that strong replicating eQTL hotspots are uncommon, at least those containing eQTLs with effect sizes that we are powered to detect with F2 cohorts of 300–500 animals.

In conclusion we believe that the data contained in this paper, including the eQTL for the four tissues– adipose, liver, kidney cortex, kidney medulla – as well the trait QTL, will be a rich source of information for the community in ongoing research to identify the genes and networks associated with metabolic and cardiovascular disease.

## Materials and Methods

### Ethics Statement

All mouse procedures were performed with the approval of Merck & Co (Whitehouse Station, NJ, USA) and the Institutional Animal Care and Use Committees at the Jackson Laboratories (Jax West, West Sacramento, California) or Mouse Clinical Institute (MCI: Strasbourg, France) as appropriate under IACUC approval 07-254.

### Mouse breeding, husbandry, and physiological trait measurements

Additional detail on mouse study design and procedures is available in the complete study protocol in Methods S1. In general, the SOPs in this study were modeled on those of the Eumorphia program (www.empress.har.mrc.uk). All procedures were performed with the approval of Merck & Co (Whitehouse Station, NJ, USA) and the Institutional Animal Care and Use Committees at the Jackson Laboratories (Jax West, West Sacramento, California) or Mouse Clinical Institute (MCI: Strasbourg, France) as appropriate.

All F2 breeding was performed at JAX West (Sacramento, CA), and knockout animals were bred at Taconic Farms (Germantown, NY). Three hundred and sixty F_2_ mice were produced from a *C57BL/6J* (female) x *A/J* (male) cross (B6AF2). F_1_ mice (n = 12 of each gender) and parental strain mice (n = 12 of each strain and gender) were tested in parallel at the same age under the same conditions. Mice were weaned into cages of three same-sex pups per litter per cross at 3 weeks of age. These three littermate mice remained together for the duration of the study. At 6 weeks, mice were shipped by air to MCI and allowed to acclimate to that environment for 1 week before entering the study. Twenty-four F_2_ mice entered MCI every week for 18 weeks, except for weeks 1, 3, and 16, when eight each of the parental and F_1_ mice entered.

An overview of the experimental protocol is provided in Figure S1. Mice were weighed after arrival at week 6, and switched to a custom high-fat high-salt balanced diet. This diet provides 60% kcal from fat, and a potassium and sodium concentration per kcal that is identical to that found in the standard D12450B diet, which provides 10% kcal from fat. All diets were prepared by Research Diets (New Brunswick, NJ). Body weight was measured weekly through week 12, with blood pressure measurement by tail cuff on a BP-2000 Blood Pressure Analysis System (Visitech Systems Inc, Apex, NC) in week 10. The mice also received a complete echocardiographic exam (Sonos 5500, Philips Electronics, Konniklijke, Netherlands) at the end of week 10 after completing the blood pressure measurements. The mice were assayed in an Oxymax metabolic chamber in week 12, underwent an overnight fast (16–18 h) followed by an Oral Glucose Tolerance Test (OGTT) in week 13, an Intra-Peritoneal Insulin Sensitization Test (IPIST) in week 14, and a Dexascan procedure in week 15. In week 16, mice were fasted for 4 hours prior to CO_2_ asphyxiation and necropsy. Detailed SOPs for these protocols are listed in the Methods S1.

### Genotyping

Genomic DNA was isolated from tail sections by Bioserve (Beltsville, MD) using standard methods and genotyping was performed by Affymetrix (Santa Clara, CA) using the Affymetrix GeneChip Mouse Mapping 5K Panel.

### Microarray analysis

RNA extraction, probe preparation, and array hybridizations were all carried out at the Rosetta Inpharmatics Gene Expression Laboratory (Seattle, WA). Mouse tissues (gonadal adipose, kidney medulla, kidney cortex, hypothalamus) were pulverized prior to homogenization in a solution of GITC/BME (1∶50 ratio) using a Covaris S2 cryo-prep (Covaris, Inc, Woburn, MA), followed by addition of a TRIzol water solution (4∶1 ratio). 100% Chloroform was added to the TRIzol/GITC lysate (1∶5 ratio) to facilitate separation of the organic and aqueous components using the phaselock (Eppendorf) system. The aqueous supernatant was further purified using a Promega SV-96 total RNA kit (Promega, Madison, WI), incorporating a DNase treatment. Total RNA samples were assayed for quality using an Agilent Bioanalyzer (Agilent Technologies, Santa Clara, CA) and for yield using Ribogreen (Ambion, Austin, TX) metrics prior to amplification. All samples, with the exception of kidney medulla, were amplified and labeled using a custom automated version of a 5 µg RT/IVT protocol and hybridizations to custom Agilent microarrays were performed as described [58].

The custom ink-jet microarrays used in this study were manufactured by Agilent Technologies and consisted of 4,732 control probes and 39,558 non-control oligonucleotides derived from mouse Unigene clusters, combined with RefSeq sequences and RIKEN full-length cDNA clones. For each individual animal tissue sample, labeled complementary RNA (cRNA) was hybridized against a pool of labeled cRNAs constructed from equal aliquots of RNA for that specific tissue from at least 200 individuals.

Gene expression profiling of *Akr1b8^−/−^, Rgs2 ^−/−^* and control wt mice was performed on Merck/Affymetrix mouse 1.0 custom arrays monitoring 43,682 individual transcripts (28,782 Entrez genes). Total RNA was isolated from frozen tissues after homogenizing in TRIzol reagent (Invitrogen, Carlsbad, CA) and processed using RNeasy kits (QIAGEN, Valencia, CA) according to manufacturers' instructions. Samples were amplified and labeled using a custom automated version of the RT/IVT protocol and reagents provided by Affymetrix (Santa Clara, CA). Hybridization, labeling and scanning were completed following the manufacturer's recommendations (Affymetrix).

All microarray data is MIAME compliant and has been deposited in GEO with the following accession number: GSE25506**.**

### QTL mapping and Network Construction

QTL mapping was performed using R/qtl [60], and testing for linkage of both clinical traits and gene expression (using the mean log ratio of the expression) was conducted using a linear model [33], [59]. Briefly, we first calculated QTL genotype probabilities, conditional on the available marker data (“calc.genoprob” function), and then used Haley-Knott regression (“scanone” function) to perform single-QTL genome scans with a normal model [60]. Specifically, each clinical and expression trait was parameterized as:
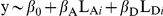
where y is the trait of interest, L_A*i*_ = P_r*i*_(AA)-P_r*i*_(BB) is the additive genotypic component at the *i*th locus and L_D*i*_ = P_r*i*_(AB) is the dominance genotypic component. For eQTL analyses, the gene expression trait values were first pre-adjusted with gender as a covariate in a linear model and the gender adjusted gene expression trait residuals used as the dependent variable in the QTL mapping. eQTL and cQTL mappings were also performed in female and male samples separately using “non-gender adjusted” values.

We conducted a permutation approach to compute False Discovery Rate (FDR) for cQTL and eQTL. LOD scores of cQTLs were empirically adjusted on a trait-by-trait basis using 1,000 permuted datasets. First, p-values were computed for each LOD score on a trait-by-trait basis, using the LOD scores from permuted data as the distribution under the null. LOD scores from the permuted data were adjusted using the same approach. FDR estimates were then computed by comparing the observed results to the permuted. Using this approach, a LOD score of 4.3 for cQTL corresponds to FDR 0.10 (Figure S13). FDR for eQTLs were computed on a tissue by tissue level using five permuted datasets. LOD scores from permuted data for all the probes were used as the distribution under the null. A LOD score of 5 for eQTL corresponds to an FDR <0.05 for all four tissues (Figure S14).

For co-expression analysis, the 9,889 (the top 25%) most differentially expressed genes were selected for constructing a weighted gene co-expression network [30]. In contrast to traditional un-weighted gene co-expression networks where two genes (nodes) are either connected or disconnected, the weighted gene co-expression network analysis assigns a connection weight to each gene pair using soft-thresholding and thus is robust to parameter selection. The weighted network analysis begins with a matrix of the Pearson correlations between all gene pairs, then converts the correlation matrix into an adjacency matrix using a power function *f(x) = x^β^*. The parameter *β* of the power function is determined in such a way that the resulting adjacency matrix, i.e., the weighted co-expression network, is approximately scale-free. To measure how well a network satisfies a scale-free topology, we use the fitting index proposed by Zhang & Horvath [30], i.e., the model fitting index *R^2^* of the linear model that regresses *log(p(k))* on *log(k)* where *k* is connectivity and *p(k)* is the frequency distribution of connectivity. The fitting index of a perfect scale-free network is 1. For this dataset, we select the smallest *β ( = 6)* which leads to an approximately scale-free network with the truncated scale-free fitting index *R^2^* greater than 0.8. The distribution *p(k)* of the resulting network approximates a power law: 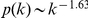.

To explore the modular structures of the co-expression network, the adjacency matrix is further transformed into a topological overlap matrix. Topological overlap between two genes reflects not only their direct interaction but also their indirect interactions through all the other genes in the network, and previous studies [30], [61] [30], [62] [28], [60]have shown that topological overlap leads to more cohesive and biologically more meaningful modules [30]. To identify modules of highly co-regulated genes, we used average linkage hierarchical clustering to group genes based on the topological overlap of their connectivity, followed by a dynamic cut-tree algorithm to dynamically cut clustering dendrogram branches into gene modules. 35 modules are identified and the module size, in number of genes, varies from 20 to 2,035.

To distinguish between modules, each module was assigned a unique color identifier, with the remaining, poorly connected genes colored grey. Figure S10 shows the hierarchical clustering over the topological overlap matrix (TOM) and the identified modules for the MCI BxA adipose. In this type of map, the rows and the columns represent genes in a symmetric fashion, and the color intensity represents the interaction strength between genes. This connectivity map highlights genes in the adipose transcriptional network that fall into distinct network modules, where genes within a given module are more interconnected with each other (blocks along the diagonal of the matrix) than with genes in other modules.

### Likelihood-based causality model selection test and causal gene enrichment test

Three basic relationships between genotypes, RNA levels and complex traits are possible once the expression of a gene (R) and a complex trait (C) have been shown to both correlated with a common QTL (L) in an F2 intercross population: where L the common locus is defined as the marker equidistant between the overlapping QTL peaks. Model M1 is a causal relationship with respect to R, in which L acts on C through transcript R. Model M2 is a reactive model with respect to R, in which R is modulated by C. Model M3 is the independent model, in which the QTL at locus L acts on traits R and C independently. M1 implies L lead to changes in trait R that in turn lead to changes in C; while M2 and M3 imply the gene expression changes lie downstream of the variation in the clinical traits or independently of the clinical traits [5]. We applied a likelihood-based causality model selection (LCMS) test that uses conditional correlation measures to determine which relationship among traits is best supported by the data [5]. The joint probability distributions corresponding to these three models, respectively, are
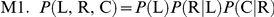


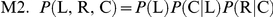


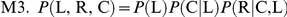
where *P*(L) is the genotype probability distribution for locus L and is based on a previously described recombination model [62]. The random variables R and C are taken to be normally distributed about each genotypic mean at the common locus L, so that the likelihoods corresponding to each of the joint probability distributions are based on the normal probability density function. Likelihoods associated with each of the models are constructed and maximized with respect to the model parameters, and the model with the smallest Akaike Information Criterion (AIC) value is identified as the model best supported by the data [63]. If M1 is selected as the best model we conclude that the data suggests that the gene expression trait is supported as causal for the clinical trait [5]. In practice we applied the LCMS test as follows. On a tissue, gender group basis we scanned the genome for cQTL and eQTLs with LOD> = 2, and then collected cQTL:eQTL genomic overlaps each time we detected peaks that overlapped at the LOD>1 threshold. These overlaps were subjected to the LCMS procedure using a marker (L) for testing selected equidistant between the overlapping QTL peaks. The statistics for these overlaps and test results are given in Table S15.

We calculated the fold enrichment for genes testing causal for a trait in the chromosome 8 eQTL signature set compared with across the genome by computing the fraction of causal reporter_ids in the chr8-eQTL signature/the fraction of causal reporter_ids on the array. The significance of the fold increase is estimated using Fisher's exact test statistics under the null hypothesis that the frequency of the causal genes in the signature set was the same between the whole reference set of genes on the array.

### Statistical Methods for eSNP Enrichment

We used a random sampling strategy to assess whether a given set of eSNPs was more likely to associate with T2D than randomly selected sets of SNPs of equal number. In each random sample, we randomly selected genotyped SNPs that were located within 1 MB of human gene regions and that had minor allele frequency (MAF) >4%, to ensure the location and MAF distributions of the random SNP sets matched that of the eSNP set of interest. The process was repeated 100,000 times. For each random SNP set, we counted the percentage of SNPs with GWAS p<0.05, 
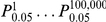
, and constructed the null distribution based on these counts. By the central limit theorem, the null distribution should approximately follow a normal distribution. This was confirmed by direct observation. We then compared the observed percentage of eSNPs with GWAS p<0.05 in the eSNP set, 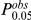, with the null distribution. We defined the Z statistic as
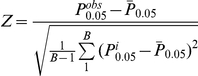
, and the p value for enrichment at the 0.05 level was thus calculated as 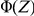, where 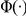 is the standard normal cumulative probability. Here we used the *p* value derived from the normal distribution rather than defining it as the percentage exceeding the observed *P* in the eSNP set from random samples (
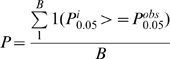
). We chose to report the theoretical *p* values to increase the resolution of our results, since over most of the testing few *p* values from the 100,000 random samples were smaller than those for the observed eSNP sets. In cases where the empirical *p* values were greater than zero, they were consistent with the theoretical *p* values.

### Methods for KO generation and phenotyping

Mice with inactivated *Akr1b8, Emr1*, and *Rgs2* genes were constructed by replacing coding regions in exon 1 of the genes (*Akr1b8*, 25 nt; *Emr1*, 71 nt; *Rgs2*, 83 nt) with a pGK-Neo cassette that would both block further transcription and cause a frame shift in any resulting mRNA. A detailed description of the *Emr1* KO generation is provided by Lin et al [64]. The *Akr1b8* and *Rgs2* null mice were provided by Deltagen (San Mateo, CA). All three KO mice strains were backcrossed to C57Blk6/N, the *Akr1b8* and *Rgs2* KO mice were backcrossed to N = 6, while the *Emr1* KO mice were backcrossed to N = 11. Mice were shipped to MCI for phenotyping. For gene expression profiling adipose tissues were collected from *Akr1b8* (n = 3) and *Rgs2* (n = 3) KO male mice and littermate controls (n = 3 per line). RNA was extracted and processed for microarray as described above. Analysis of microarray data was performed using a one-way ANOVA to compare the genotypic groups (KO v WT) following normalization of the data with the RMA algorithm. The results of this analysis are available in Table S16. Enrichment analysis of the KO signatures versus the MCI BxA F2 adipose co-expression modules (Figure S10) was performed using Entrez Gene IDs (LLIDs) to match across platforms (Affymetrix to Agilent). Unique IDs for each category were tallied and fold-enrichment calculated with significance derived from the hypergeometric *P* value (Table S14).

## Supporting Information

Figure S1 Phenotyping platform and timeline for trait collection in the B6AF2 cohort. - Mice were shipped from USA to France at 6 weeks of age, acclimatized for 1 week and then entered the study. Mice were fed a modified high fat, salt balanced diet for the duration of the study (9 weeks), and were subjected to phenotying at the illustrated times. Details on the individual trait measures are included in the methods and supplemental methods. Mice were sacrificed at 16 weeks and tissues harvested for gene expression profiling.
(0.43 MB TIF)

Figure S2 Adiposity QTL hotspot on chromosome 8 coincides with QTL for Energy and Glucose Traits. - QTL plots showing adiposity, energy, and glucose traits mapping to chromosome 8. Note that not all traits are shown for clarity. For additional information on QTLs at this locus see Supplemental Table S2.
(0.53 MB TIF)

Figure S3 Hierarchical clustering of traits across the mouse F2 population. - Trait values from the 360 individuals from the F2 population were normalized to allow comparison by converting to Z-scores. They were hierarchically clustered using an UPGMA unweighted average. Individual mice are represented as rows and traits as columns. Red represents Z>2 and green Z<-2.
(1.60 MB TIF)

Figure S4 eQTL tissue distributions and characterization of cis versus trans eQTL. - (A) cis and trans eQTL LOD distribution across tissues; (B) counts of eQTL (Y-axis) versus distance in bp between gene and QTL position (X-axis) for genes that are physically located on a different chromosome from the eQTL (gray) or on the same chromosome as the eQTL (black).
(1.21 MB TIF)

Figure S5 eQTL chromosomal distribution by tissue - adipose (A), kidney cortex (B), kidney medulla (C), liver (D). - The count indicated on the Y-axis refers to the number of unique reporter_ids. Note the peak of eQTLs in the middle portion of chromosome 8 specific to the adipose tissue (A). Other eQTL hotpsots are apparent, notably a liver-specific eQTL signature on chromosome 6.
(1.18 MB TIF)

Figure S6 Frequency of eQTL Hotspots in Observed and Permuted Data. The figure shows a frequency distribution for the number of times a 10 Mb genomic bin contains a certain number of eQTLs with LOD>5 (X-axis) for the observed and permuted data from adipose. All four of the 10 Mb bins containing >1000 eQTLs in the observed data are from chromosome 8. The maximum number of eQTLs seen in any 10 Mb bin across the permuted data is 38 in Permute set 1.
(0.14 MB TIF)

Figure S7 Plot of the MCI BxA adipose LOD scores versus the Jaxshort BxA adipose LOD scores for trans eQTL from the chromosome 8 hotspot. Shown are gene reporters that map to the 30-40 cM interval on chromosome 8 in the MCI BxA cohort and the maximum LODs for the corresponding reporters in the Jaxshort BxA cohort on chromosome 8.
(0.15 MB TIF)

Figure S8 QTL maximum position on chromosome 8 for the replicating trans eQTL in the Jaxshort BxA cross. This figure illustrates that most of the eQTL map to a similar position (30–40 cM interval) on chromosome 8.
(0.08 MB TIF)

Figure S9 Mouse Trans8_eQTL signature is enriched in GO Biological Process terms associated with energy metabolism, oxidative phosphorylation and mitochondrial function. - The hierarchical structure represents the relationship between GO biological terms in the human Gene Ontology. The terms are colored according to the degree of enrichment for genes in the trans8_eQTL signature (red, P∼10-30: mid pink, P∼10-10: light pink, P∼10-6). Only terms with statistically significant enrichments are shown (P<10-6).
(1.73 MB TIF)

Figure S10 Mouse Trans8_eQTL signature maps to two modules in the adipose co-expression network. - This figure shows the topological overlap map for adipose from the MCI BxA cohort. The trans8_eQTL signature is highly enriched in the red and turquoise modules. These modules are highly correlated with metabolic traits and associated with GO terms for mitochondria and metabolic processes (red) and angiogenesis, apoptosis, cell cycle, and immune cell function (turquoise).
(1.68 MB TIF)

Figure S11 Mouse Trans8_eQTL signature maps to human adipose modules whose expression correlates with metabolic traits. - The human male adipose connectivity map is as previously described [25]. The enrichment P values for the overlap of the mouse trans8_eQTL signature to the red and turquoise modules are given as well as the traits with which the modules correlate. Gene ontology annotation of the genes in these modules is also shown.
(1.11 MB TIF)

Figure S12 SNP Set P value Distribution from DIAGRAM GWAS. - The binned P values are shown on the X-axis for (A) the full set of all SNPs with MAF >4% and (B) the set of 6,720 eSNPs associated with adipose gene expression in the trans8_eQTL signature.
(0.72 MB TIF)

Figure S13 FDR by LOD score plot for cQTL.
(0.00 MB PNG)

Figure S14 FDR by LOD score plot for eQTL for Four Tissues; Liver, Adipose, Kidney Medulla, Kidney Cortex.
(0.00 MB PNG)

Table S1 Trait values across the Parental, F1 and F2 cohorts.
(0.03 MB XLS)

Table S2 Trait QTL identified in the B6AF2 Cohort.
(0.18 MB XLS)

Table S3 eQTL identified in the B6AF2 Cohort using All genders.
(5.93 MB XLS)

Table S4 eQTL identified in the B6AF2 Cohort using Male only.
(3.59 MB XLS)

Table S5 eQTL identified in the B6AF2 Cohort using Female only.
(3.84 MB XLS)

Table S6 cis and trans eQTL sharing between tissues for eQTLs LOD> = 5.
(0.02 MB XLS)

Table S7 Corrected (batch and gender) Gene Expression Data for 1722 reporter_ids representing the chromosome 8 hotspot in the Jaxshort BxA Adipose Replication Cohort.
(4.35 MB CSV)

Table S8 Genotype Data for the Jaxshort BxA Replication Cohort.
(2.84 MB CSV)

Table S9 Gender assignments for Jaxshort BxA replication cohort.
(0.04 MB XLS)

Table S10 Enrichment Analysis for causal genes in trans8_eQTL signature.
(0.02 MB XLS)

Table S11 Body Weight data for Akr1b8-/-, Emr1-/-, and Rgs2-/- and littermate controls.
(0.03 MB XLS)

Table S12 Body Composition data by qNMR for Akr1b8-/-, Emr1-/-, and Rgs2-/- and littermate controls.
(0.07 MB XLS)

Table S13 Serum Lipid composition for Akr1b8-/-, Emr1-/-, and Rgs2-/- and littermate controls.
(0.05 MB XLS)

Table S14 Enrichment of Akr1b8-/- and Rgs2-/- adipose signatures in the MCI BxA F2 adipose co-expression modules associated with the trans eQTL signature and adiposity traits.
(0.02 MB XLS)

Table S15 Number of cQTL:eQTL pairs tested for causal/reactive/independent/no call relationship grouped by Tissue and Gender.
(0.03 MB XLS)

Table S16 ANOVA Analysis of Akr1b8-/- (n = 3) and Rgs2-/- (n = 3) adipose signatures versus littermate controls.
(7.64 MB XLS)

Methods S1 (0.07 MB DOC)
