# Supplementary figures and images for: Identification of Genes and Networks Driving Cardiovascular and Metabolic Phenotypes in a Mouse F2 Intercross
Source: PLoS One. 2010 Dec 14;5(12):e14319. doi: 10.1371/journal.pone.0014319 (PMC3001864; doi:10.1371/journal.pone.0014319)

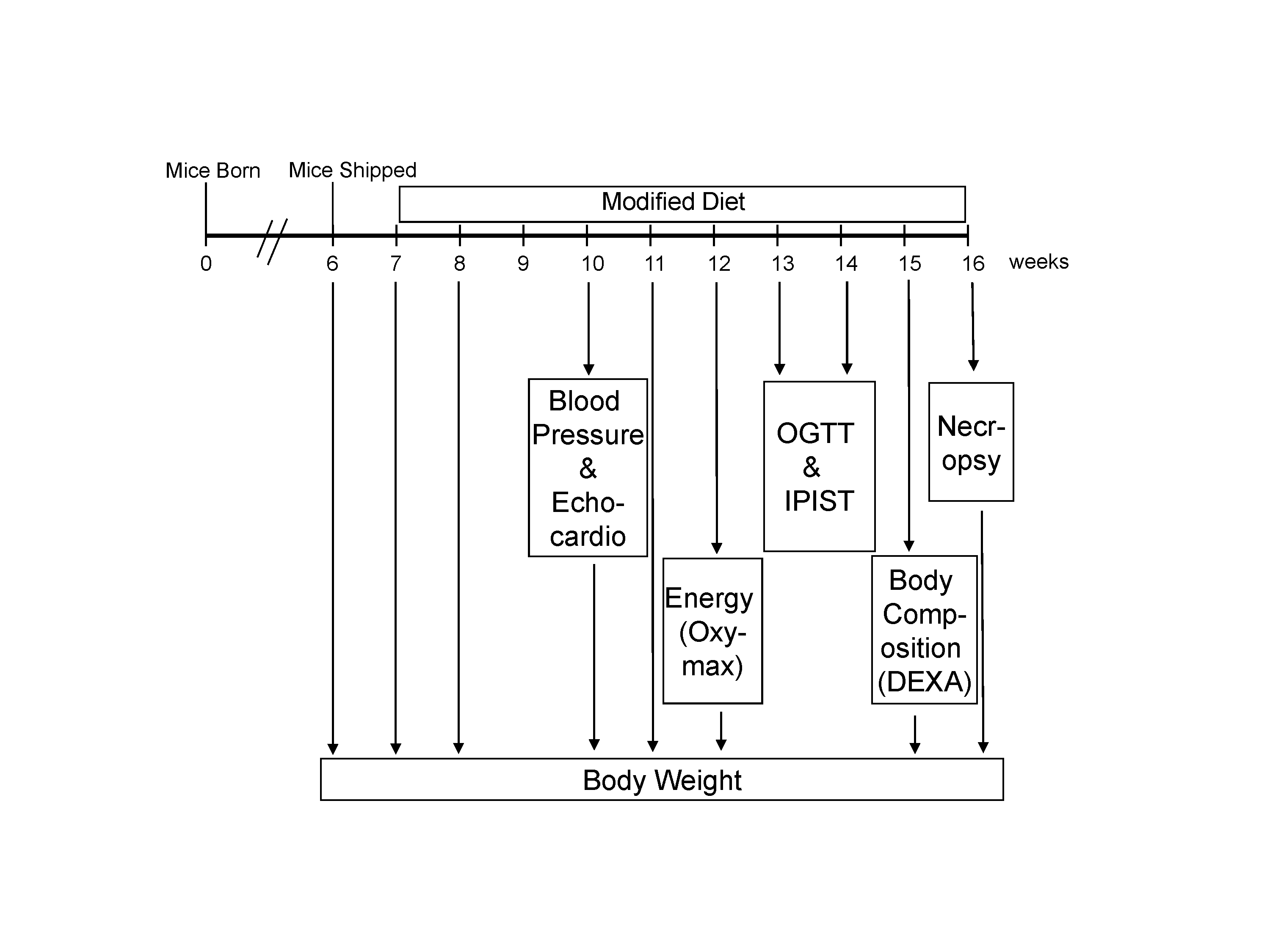

Supplement: Figure S1 — Phenotyping platform and timeline for trait collection in the B6AF2 cohort. - Mice were shipped from USA to France at 6 weeks of age, acclimatized for 1 week and then entered the study. Mice were fed a modified high fat, salt balanced diet for the duration of the study (9 weeks), and were subjected to phenotying at the illustrated times. Details on the individual trait measures are included in the methods and supplemental methods. Mice were sacrificed at 16 weeks and tissues harvested for gene expression profiling. (0.43 MB TIF) [file pone.0014319.s001.tif]

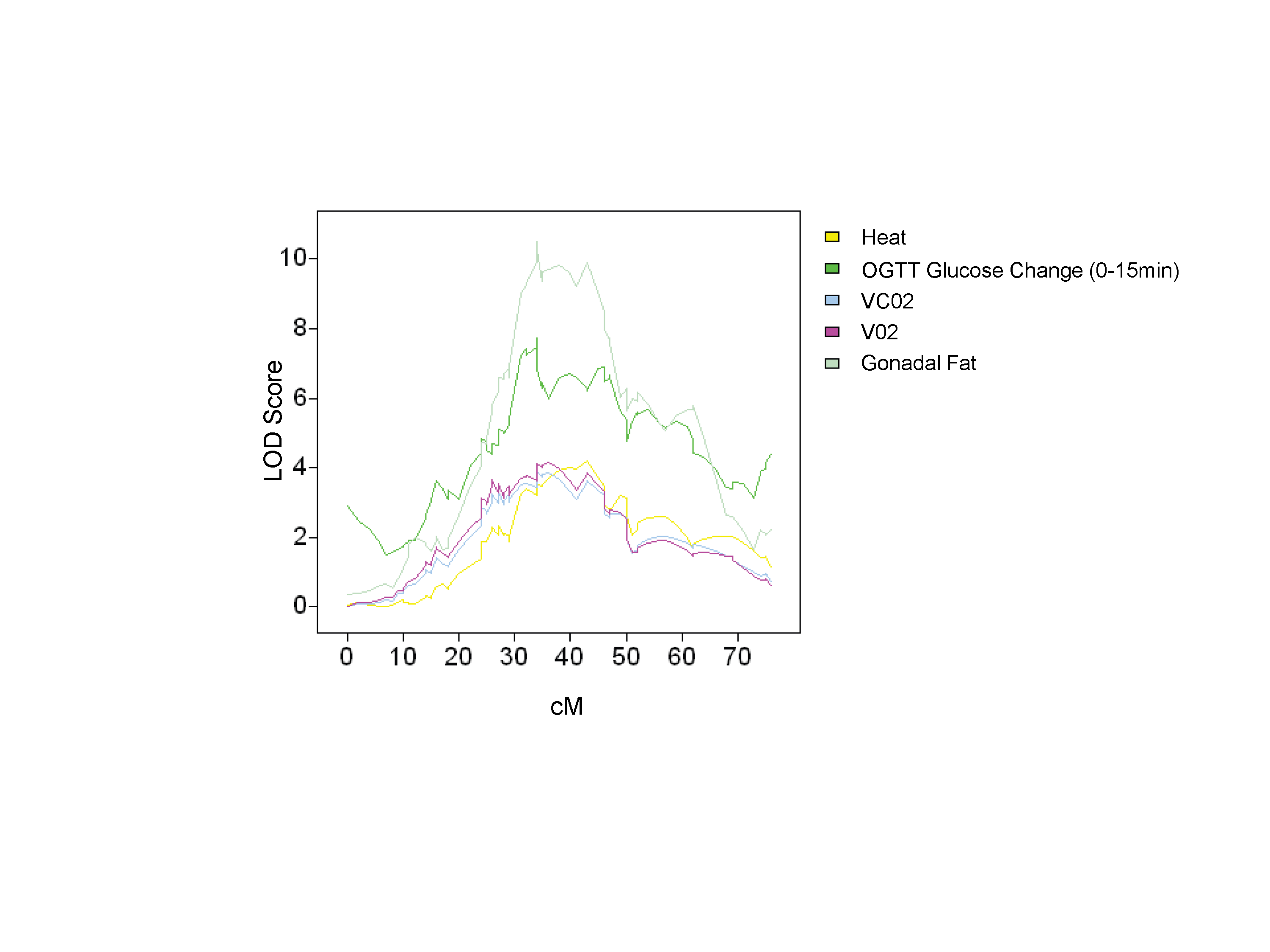

Supplement: Figure S2 — Adiposity QTL hotspot on chromosome 8 coincides with QTL for Energy and Glucose Traits. - QTL plots showing adiposity, energy, and glucose traits mapping to chromosome 8. Note that not all traits are shown for clarity. For additional information on QTLs at this locus see Supplemental Table S2. (0.53 MB TIF) [file pone.0014319.s002.tif]

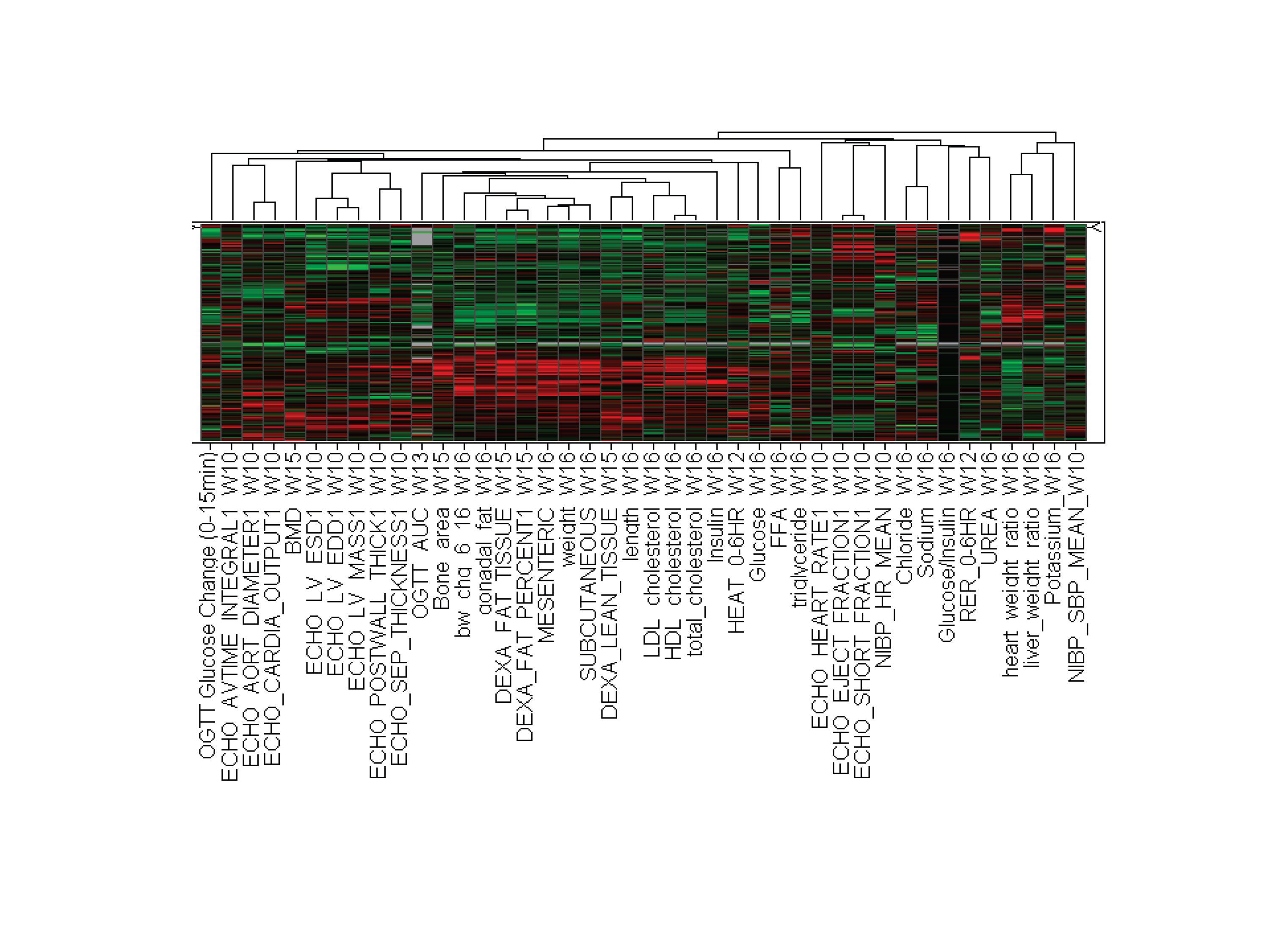

Supplement: Figure S3 — Hierarchical clustering of traits across the mouse F2 population. - Trait values from the 360 individuals from the F2 population were normalized to allow comparison by converting to Z-scores. They were hierarchically clustered using an UPGMA unweighted average. Individual mice are represented as rows and traits as columns. Red represents Z>2 and green Z<-2. (1.60 MB TIF) [file pone.0014319.s003.tif]

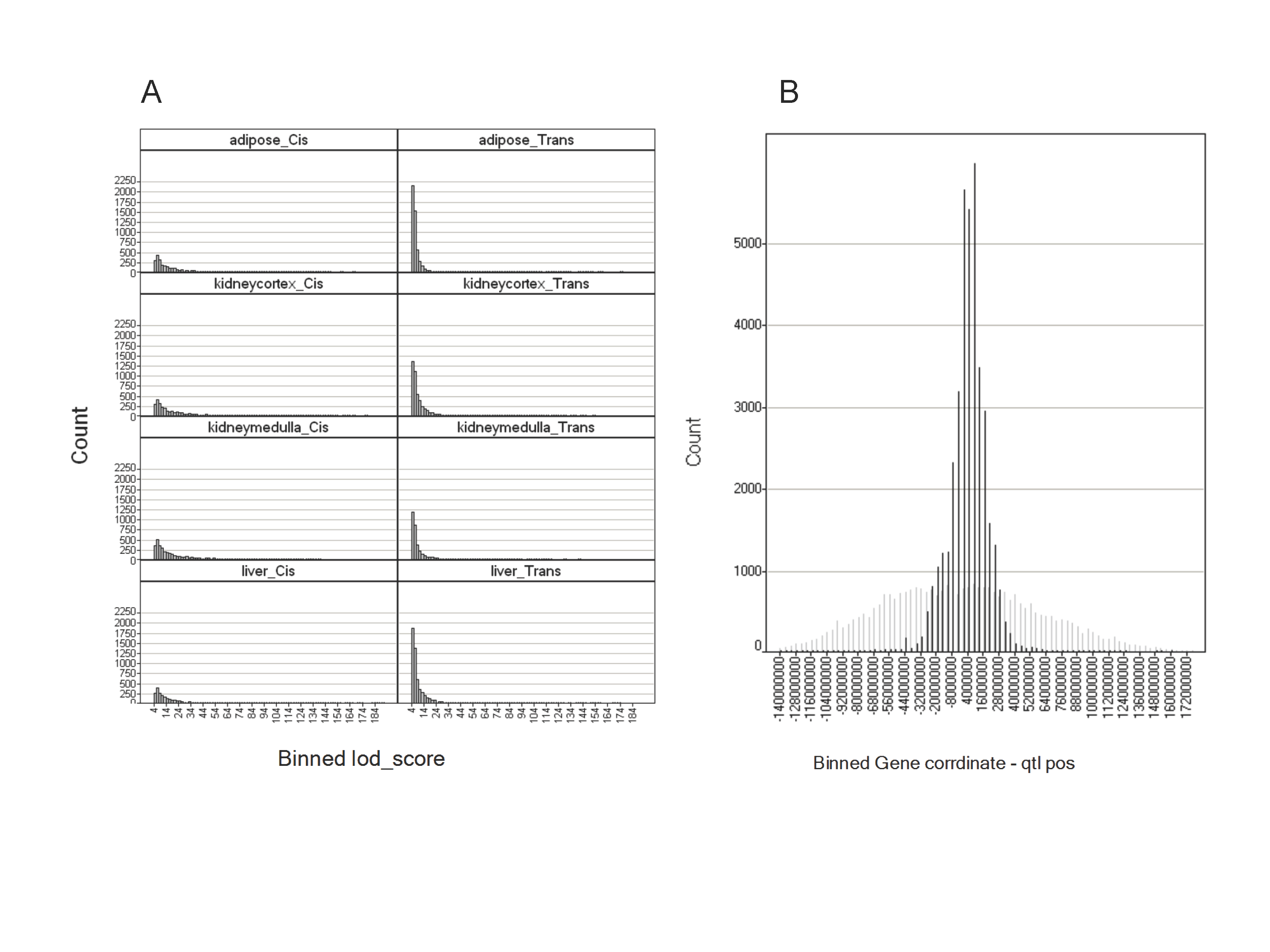

Supplement: Figure S4 — eQTL tissue distributions and characterization of cis versus trans eQTL. - (A) cis and trans eQTL LOD distribution across tissues; (B) counts of eQTL (Y-axis) versus distance in bp between gene and QTL position (X-axis) for genes that are physically located on a different chromosome from the eQTL (gray) or on the same chromosome as the eQTL (black). (1.21 MB TIF) [file pone.0014319.s004.tif]

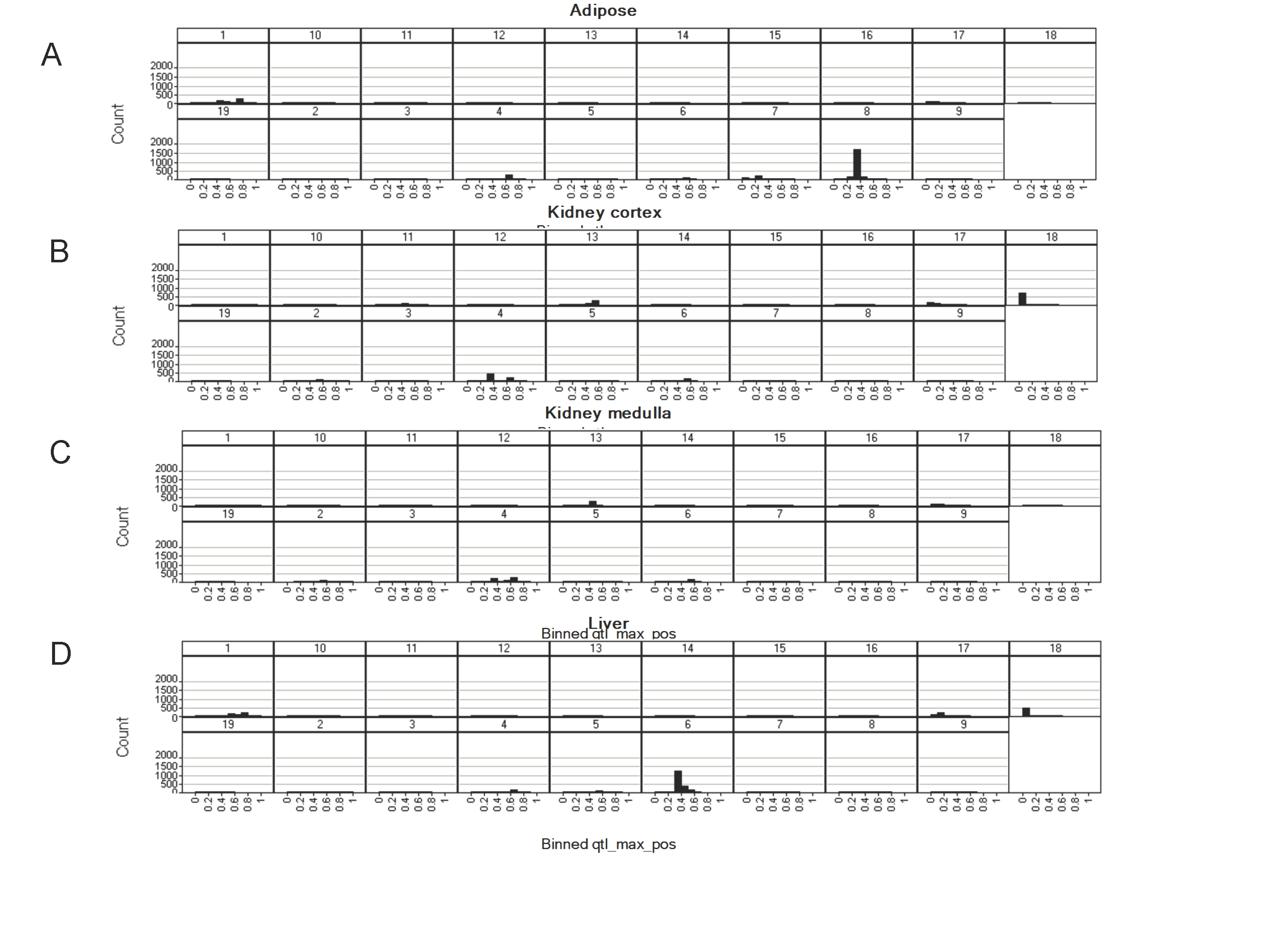

Supplement: Figure S5 — eQTL chromosomal distribution by tissue - adipose (A), kidney cortex (B), kidney medulla (C), liver (D). - The count indicated on the Y-axis refers to the number of unique reporter_ids. Note the peak of eQTLs in the middle portion of chromosome 8 specific to the adipose tissue (A). Other eQTL hotpsots are apparent, notably a liver-specific eQTL signature on chromosome 6. (1.18 MB TIF) [file pone.0014319.s005.tif]

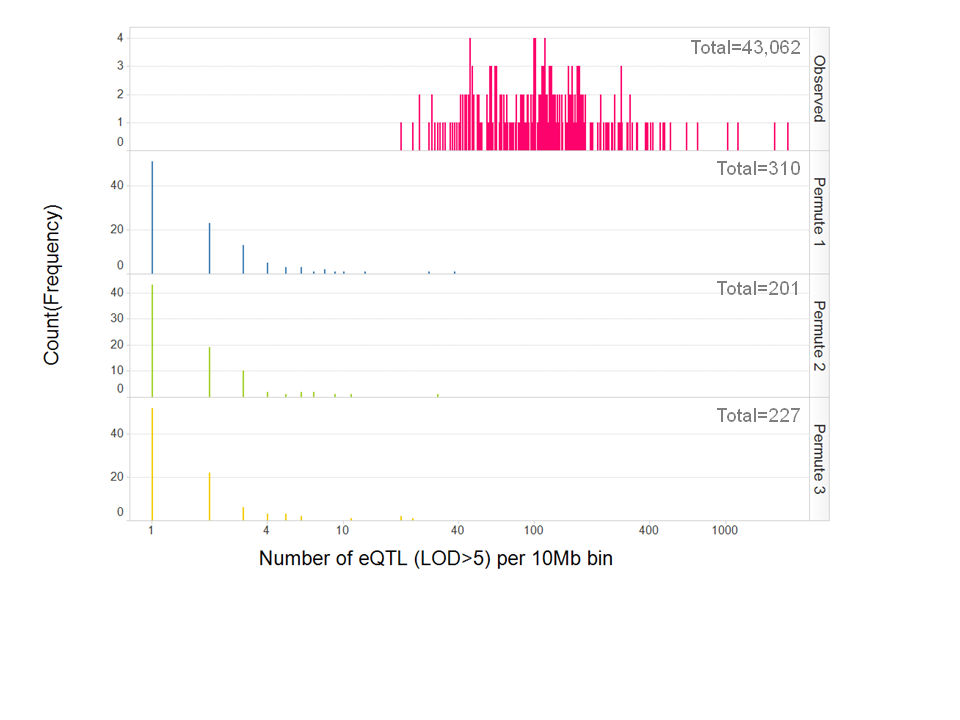

Supplement: Figure S6 — Frequency of eQTL Hotspots in Observed and Permuted Data. The figure shows a frequency distribution for the number of times a 10 Mb genomic bin contains a certain number of eQTLs with LOD>5 (X-axis) for the observed and permuted data from adipose. All four of the 10 Mb bins containing >1000 eQTLs in the observed data are from chromosome 8. The maximum number of eQTLs seen in any 10 Mb bin across the permuted data is 38 in Permute set 1. (0.14 MB TIF) [file pone.0014319.s006.tif]

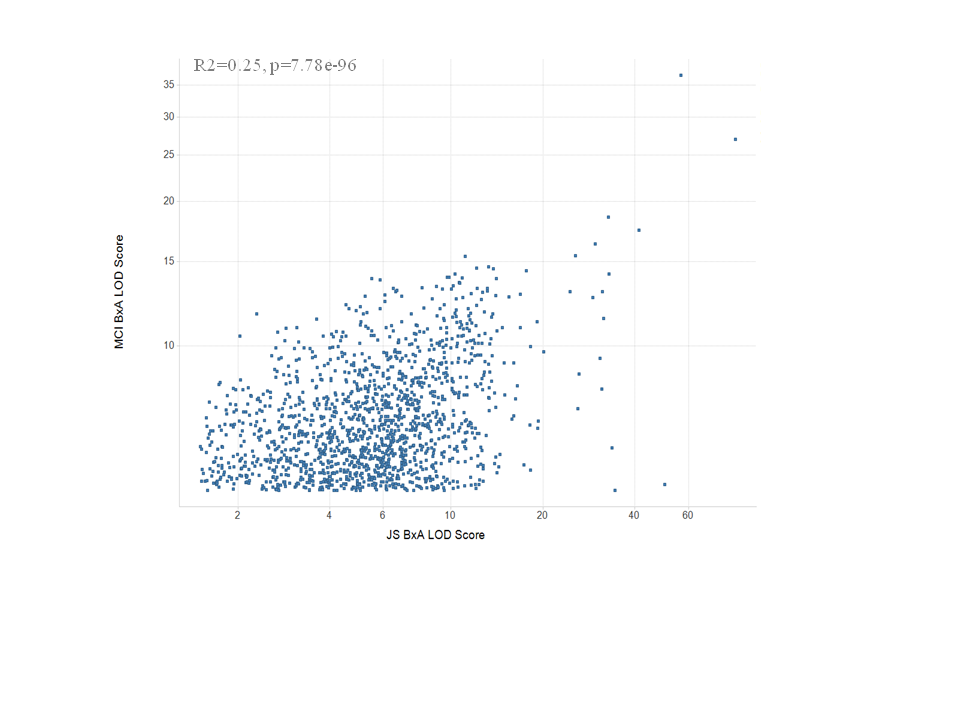

Supplement: Figure S7 — Plot of the MCI BxA adipose LOD scores versus the Jaxshort BxA adipose LOD scores for trans eQTL from the chromosome 8 hotspot. Shown are gene reporters that map to the 30-40 cM interval on chromosome 8 in the MCI BxA cohort and the maximum LODs for the corresponding reporters in the Jaxshort BxA cohort on chromosome 8. (0.15 MB TIF) [file pone.0014319.s007.tif]

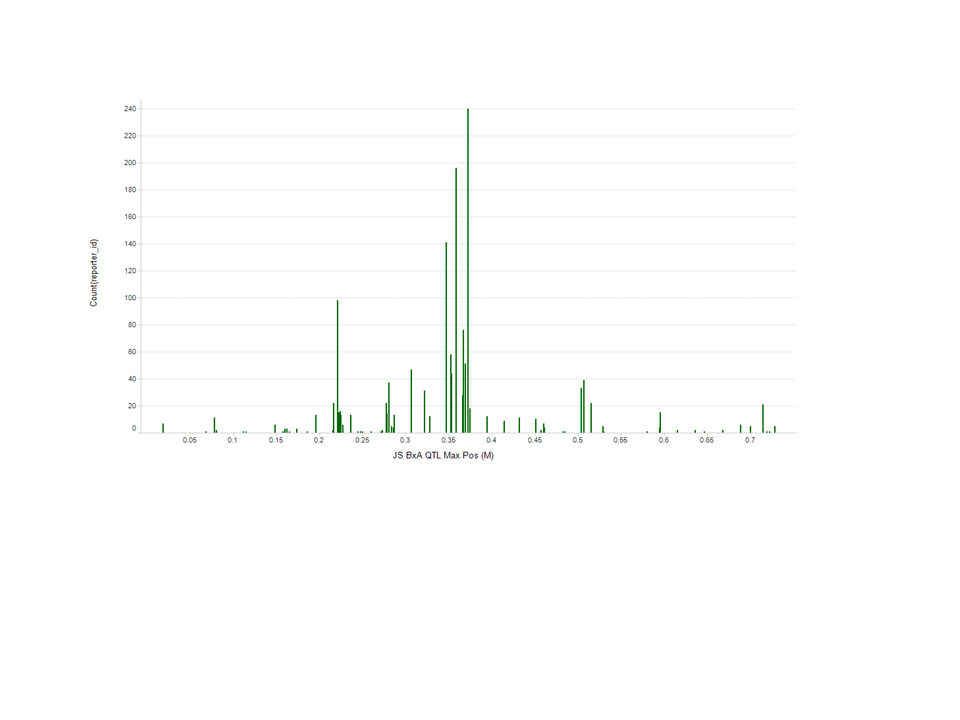

Supplement: Figure S8 — QTL maximum position on chromosome 8 for the replicating trans eQTL in the Jaxshort BxA cross. This figure illustrates that most of the eQTL map to a similar position (30–40 cM interval) on chromosome 8. (0.08 MB TIF) [file pone.0014319.s008.tif]

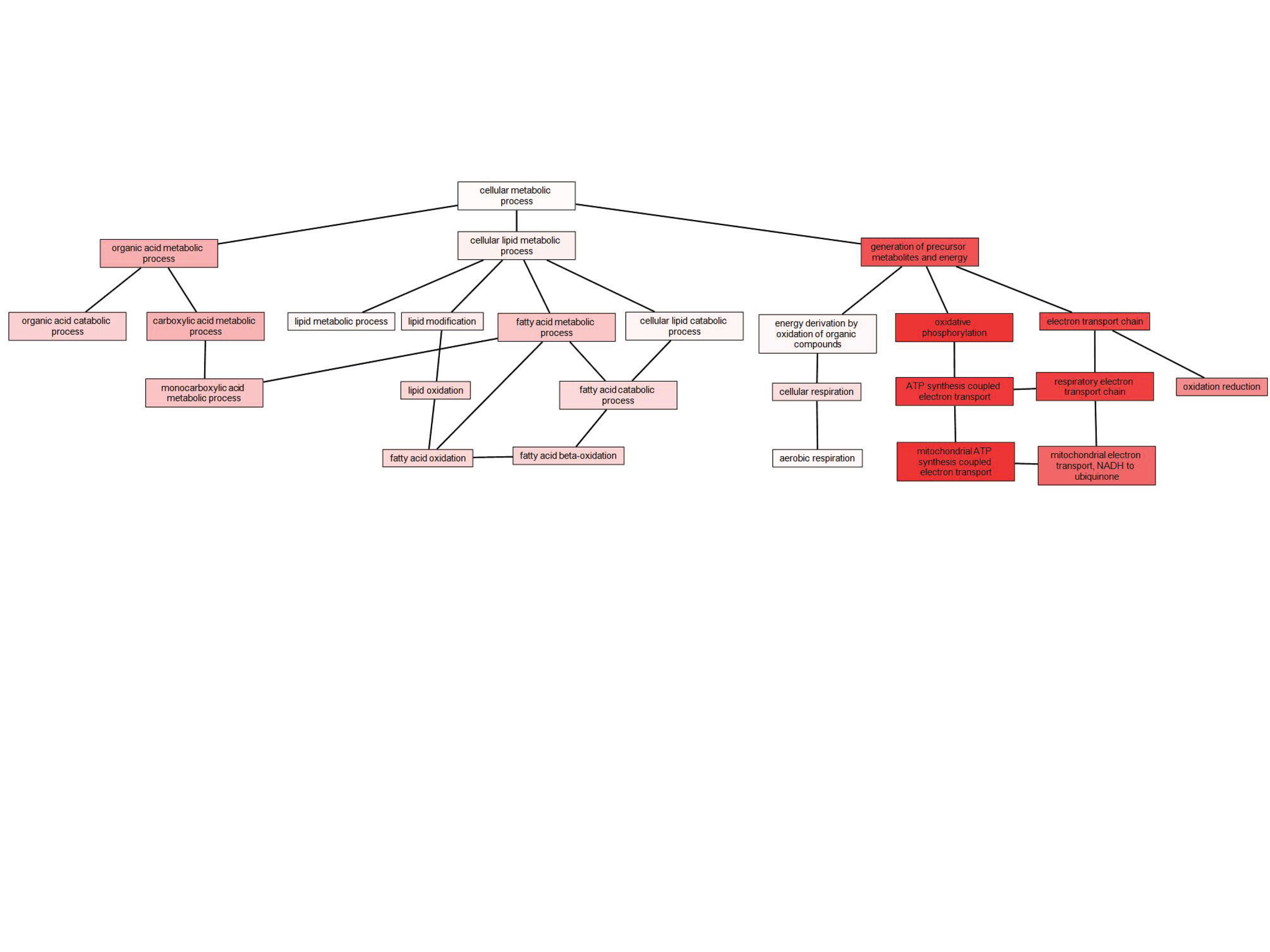

Supplement: Figure S9 — Mouse Trans8_eQTL signature is enriched in GO Biological Process terms associated with energy metabolism, oxidative phosphorylation and mitochondrial function. - The hierarchical structure represents the relationship between GO biological terms in the human Gene Ontology. The terms are colored according to the degree of enrichment for genes in the trans8_eQTL signature (red, P∼10-30: mid pink, P∼10-10: light pink, P∼10-6). Only terms with statistically significant enrichments are shown (P<10-6). (1.73 MB TIF) [file pone.0014319.s009.tif]

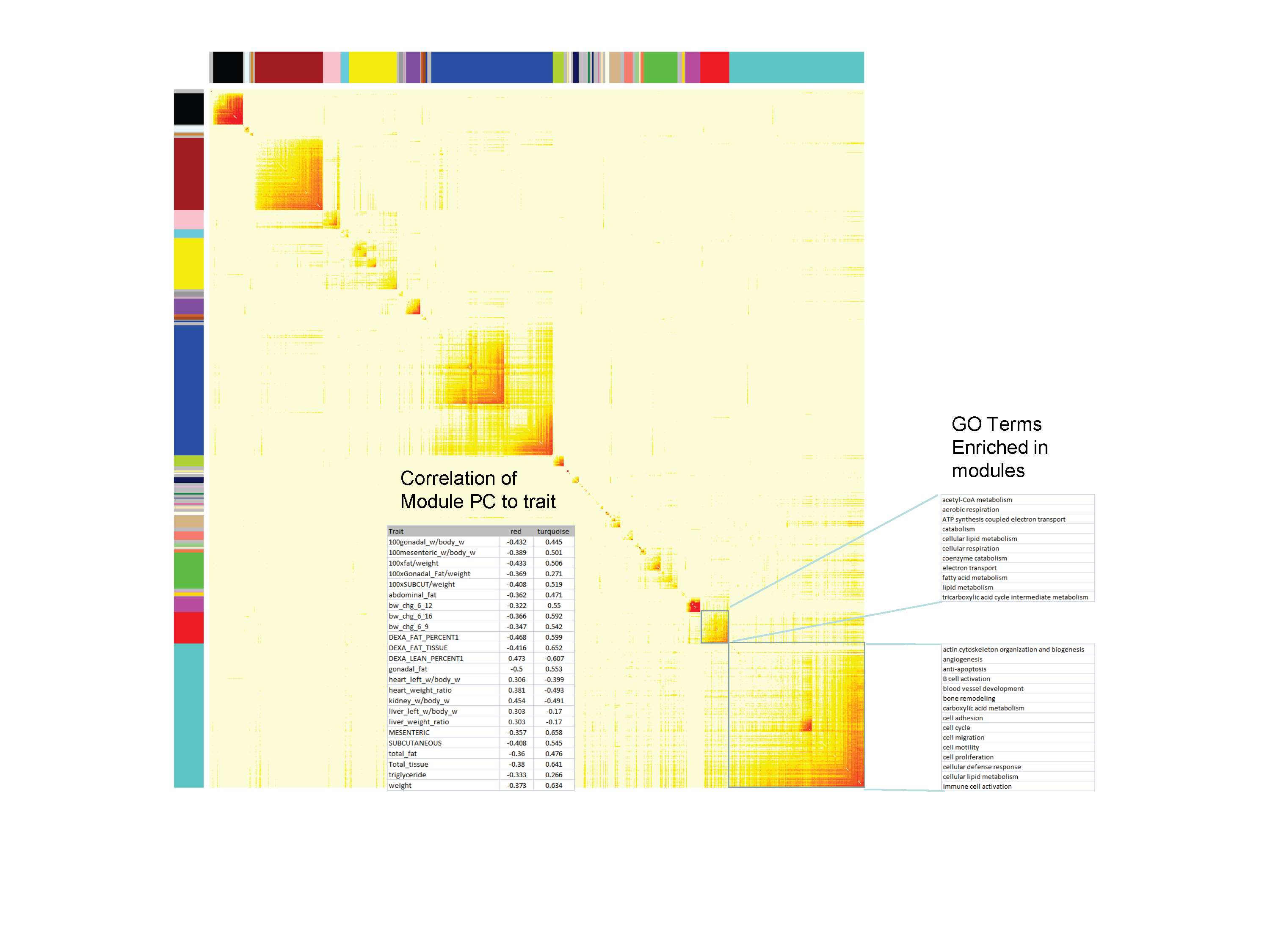

Supplement: Figure S10 — Mouse Trans8_eQTL signature maps to two modules in the adipose co-expression network. - This figure shows the topological overlap map for adipose from the MCI BxA cohort. The trans8_eQTL signature is highly enriched in the red and turquoise modules. These modules are highly correlated with metabolic traits and associated with GO terms for mitochondria and metabolic processes (red) and angiogenesis, apoptosis, cell cycle, and immune cell function (turquoise). (1.68 MB TIF) [file pone.0014319.s010.tif]

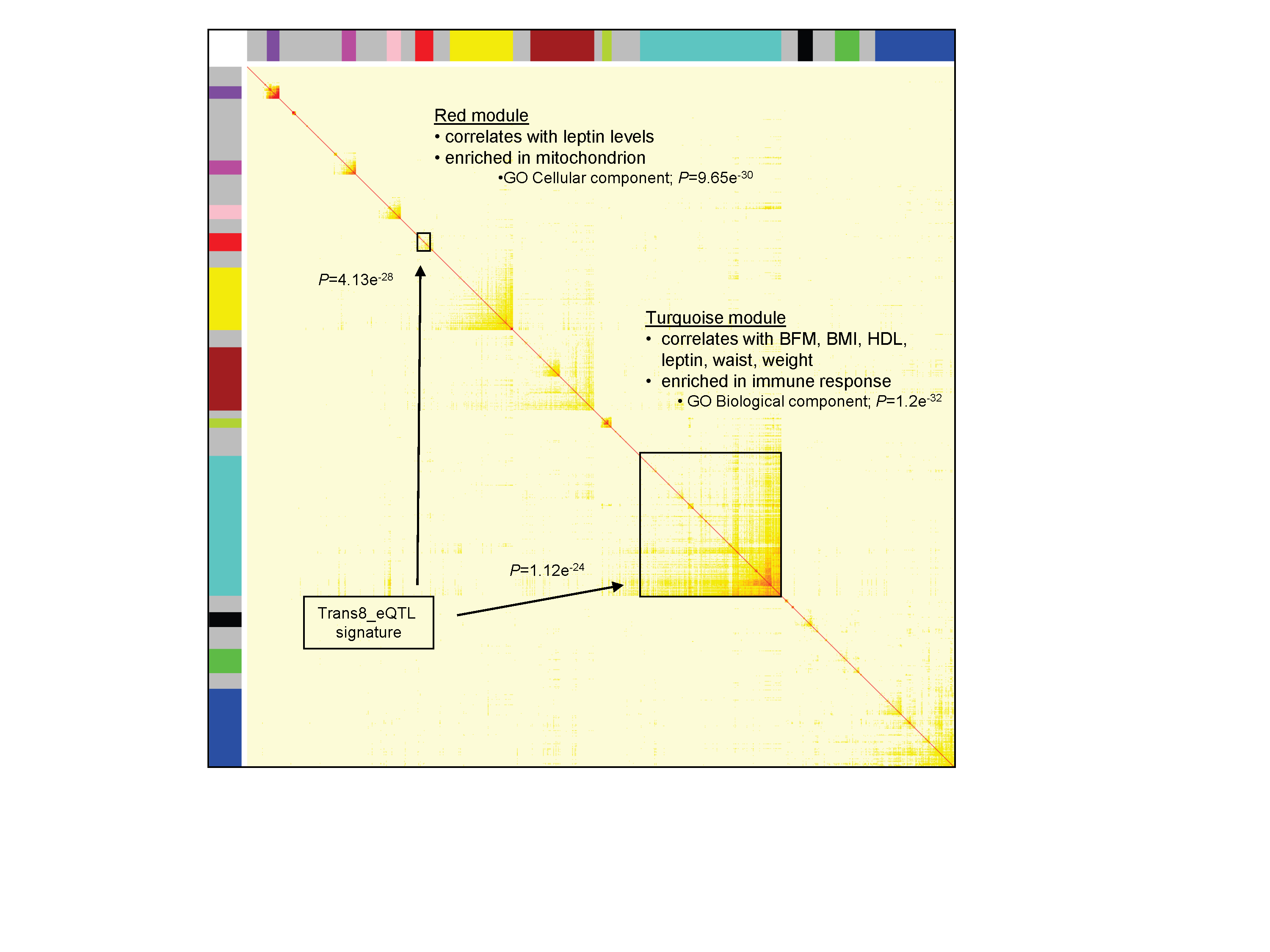

Supplement: Figure S11 — Mouse Trans8_eQTL signature maps to human adipose modules whose expression correlates with metabolic traits. - The human male adipose connectivity map is as previously described [25]. The enrichment P values for the overlap of the mouse trans8_eQTL signature to the red and turquoise modules are given as well as the traits with which the modules correlate. Gene ontology annotation of the genes in these modules is also shown. (1.11 MB TIF) [file pone.0014319.s011.tif]

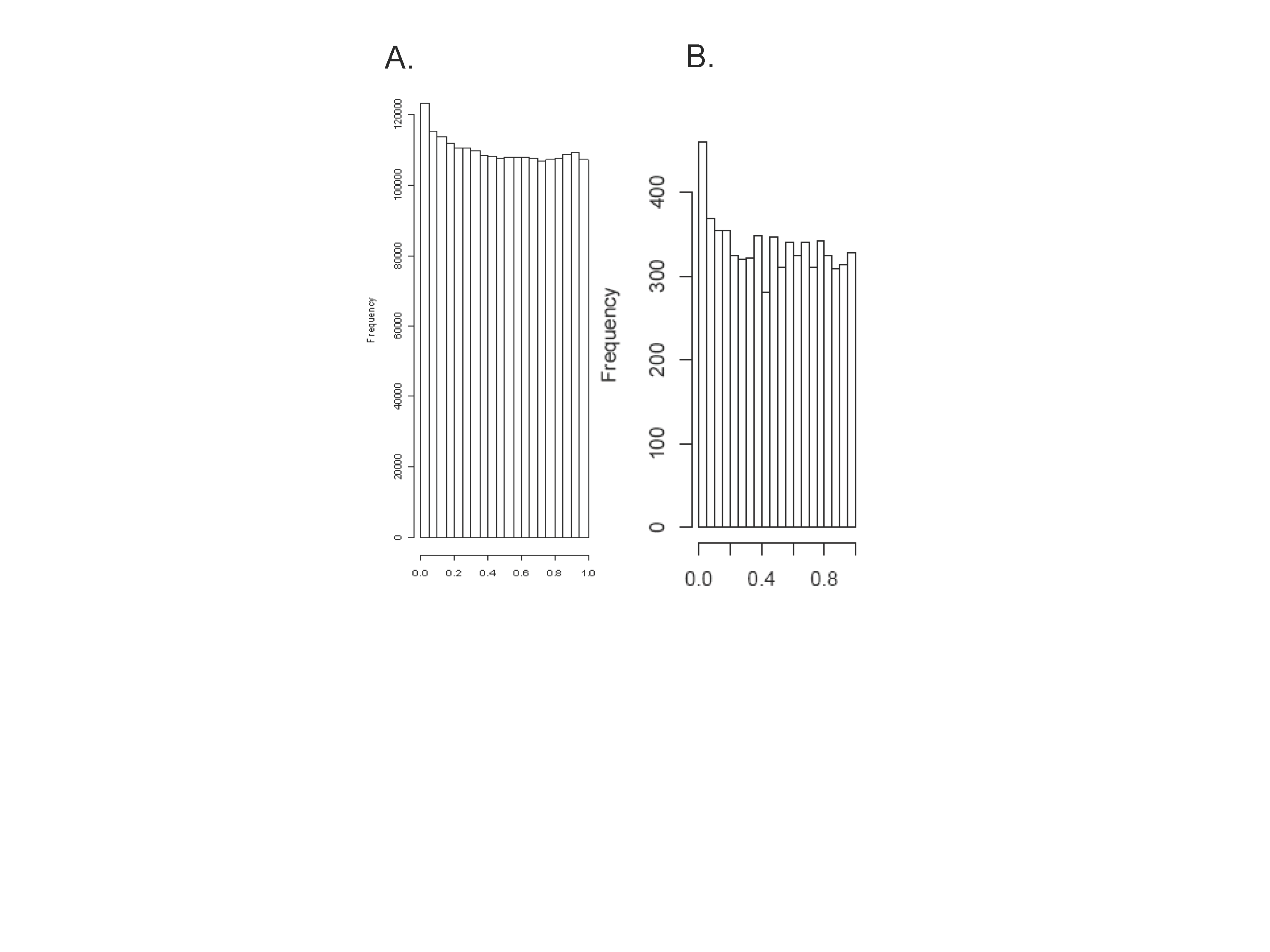

Supplement: Figure S12 — SNP Set P value Distribution from DIAGRAM GWAS. - The binned P values are shown on the X-axis for (A) the full set of all SNPs with MAF >4% and (B) the set of 6,720 eSNPs associated with adipose gene expression in the trans8_eQTL signature. (0.72 MB TIF) [file pone.0014319.s012.tif]

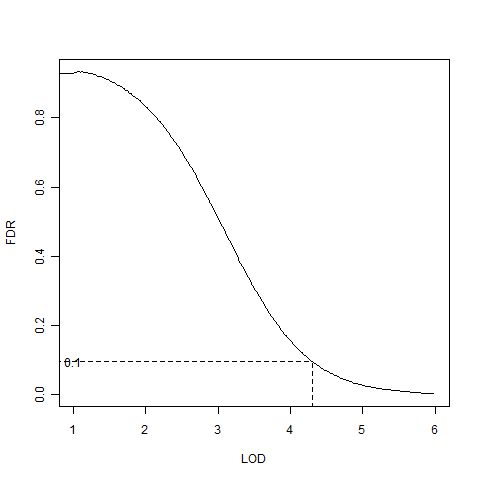

Supplement: Figure S13 — FDR by LOD score plot for cQTL. (0.00 MB PNG) [file pone.0014319.s013.png]

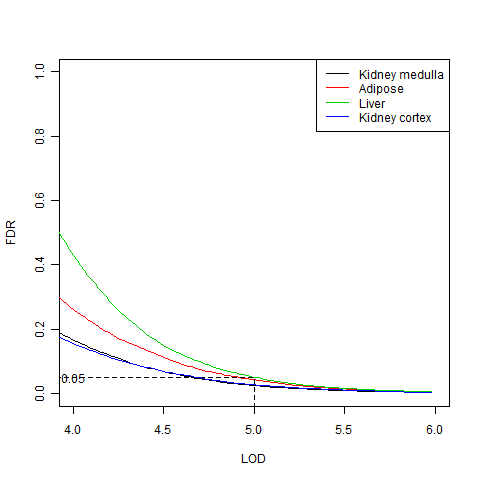

Supplement: Figure S14 — FDR by LOD score plot for eQTL for Four Tissues; Liver, Adipose, Kidney Medulla, Kidney Cortex. (0.00 MB PNG) [file pone.0014319.s014.png]
