# Supplementary material for: Identification of Genes and Networks Driving Cardiovascular and Metabolic Phenotypes in a Mouse F2 Intercross
Source: PLoS One. 2010 Dec 14;5(12):e14319. doi: 10.1371/journal.pone.0014319 (PMC3001864; doi:10.1371/journal.pone.0014319)
Supplement: Methods S1 — (0.07 MB DOC) [file pone.0014319.s031.doc]

**Supplemental methods**

**Design Considerations**

Experimental design was optimized to minimize confounding variables. Specifically, mice entered the study at a rate of 12 females and 12 males per week, with a rest week every 5^th^ week during which no new mice entered the study protocol. We staggered entry dates for F_2_ mice from all crosses, parental strains, and F_1_ strains to minimize any confounding of genotype by calendar time of procedures. Furthermore, all assays were performed within narrow time windows during the day to minimize the impact of circadian effects on the data. For the hypertension assays mice underwent all protocol procedures in random order. We randomly assigned location of cages within the rack in the mouse room, and cage location remained the same throughout the study. Random order was initially assigned by cage, and then all three mice in the cage underwent that procedure in random order based on whichever mouse was caught first.

**SOPs**

Body weights

Body weights were recorded once a week, on the same day every week, from the age of 6 weeks to 12 weeks.

Blood pressure

Heart rate and systolic blood pressure were measured in conscious animals by a computerized tail-cuff system (BP-2000, Visitech Systems, Apex, NC). Mice were trained with 10 consecutive preliminary measurements that were then followed by 10 study measurements that were recorded and averaged for each individual animal. The procedure was considered successful when 7 out of the 10 measurements were valid with a standard deviation <10mmHg. Recordings were performed in the morning on five consecutive days. The mean of these five measurements were averaged and used for statistical comparisons.

Transthoracic Echocardiography

Animals were weighed and analyzed for cardiac anatomy and function on a Sonos 5500 (Philips Electronics, Koninklijke, The Netherlands) with a 15-MHz linear transducer (15L6). All examinations were performed in mice anesthetized with sodium pentobarbital (30 mg/kg IP). The following parameters were measured: 1) left ventricular end-diastolic and end systolic diameters (EDD and ESD), septal and posterior wall (SW and PW) thicknesses, and left ventricular mass (LVM= 1.055x[(EDD+SW+PW)^3^-EDD^3^)]) to assess cardiac anatomy; 2) left ventricular fractional shortening (FS= [(EDD-ESD)/ESD]x100), ejection fraction (EF), and cardiac output (CO) to assess systolic function. CO was obtained by placing the Doppler window into the ascending aorta on a long-axis view and applying a fixed 60° angular correction (CO= 0.785 x Dao^2^ x VTI x HR, where Dao is the aorta diameter and VTI the velocity time integral of the aortic flow obtained by pulsed Doppler). Except for this last method, all measurements were performed according to the guidelines of the American Society of Echocardiography.

Energy expenditure

Energy expenditure was evaluated by indirect calorimetry using the Oxymax apparatus (Columbus Instrument-Columbus, OH). Briefly, the system is designed to monitor oxygen consumption and production of carbon dioxide and heat; at the same time activity is evaluated using the infra-red beam break. The total duration of the experiment was approximately 19-20 hours. Mice were put in individual cages and VO_2_ and VCO_2_ measurements were recorded every 26 minutes. Oxymax provided VO_2_ and VCO_2_ figures in the units ml/kg^0.75^/hr. The respiratory exchange ratio (RER) is calculated as VCO_2_/VO_2_. Heat (Kcal/ kg^0.75^/hr) is calculated from the VO_2_ and RER information using the standard formula: Heat (H) = (Calorific Value x VO_2_ x 0.001) / body weight^0.75^, where Calorific value (CV) = 3.815 + 1.232xRER. The test was performed at thermo-neutrality (30°C).

Oral glucose tolerance test (OGTT)

OGTT was performed on mice that were fasted overnight. After measurement of the basal glucose level using a drop of blood collected from the tail (time 0), mice were injected per os with a solution of 20% glucose in sterile saline (0.9% NaCl) at a dose of 2g glucose/kg body weight. Blood was collected from the tail 15, 30, 45, 60, 90, 120, 150, 180 min after injection for glucose determination. The incremental areas of the glucose curve (AUC) from T0-T180 min, and the % glucose increase from T0-T15min were calculated.

Intra-peritoneal insulin sensitivity test (IPIST)

The blood glucose-lowering effect of insulin was assessed by the intraperitoneal insulin sensitivity test. Briefly, overnight fasted animals were given an intraperitoneal injection of 0.2 mg/ml pork insulin solution (Sigma) at a dose of 0.5 UI/kg body weight. Blood was collected 0, 15, 30, 45, 60, and 90 min after insulin injection and glucose measured by a glucometer (Accu-check Active blood glucose monitor, Roche Diagnostics). The % glucose change from T0-T15 min was also calculated.

Dexascan analysis

Body composition (lean and fat content) and bone mineral density were evaluated by dual-energy X-ray analysis on an ultrahigh-resolution densitometer PIXImus (GE Medical Systems). Mice were anesthetized by intraperitoneal injection of ketamine/xylazine solution and then placed on the specimen tray. Bone mineral density (BMD; in g/cm^2^), bone mineral content (BMC; in g), bone area (in cm^2^), and body composition (fat and lean tissue in % and in g) were recorded. The total acquisition time was less than 5 minutes.

Blood sample collection and analysis

After 4 hours fasting, blood was collected by intracardiac puncture after CO_2_ asphyxiation. Approximately 300 µl of blood was collected into a heparinized tube for biochemical analysis. Blood sampling was performed as described previously (Champy et al. 2004).

Eleven biochemical parameters were measured including electrolytes (sodium, potassium, and chloride), metabolites (glucose, urea, creatinine, total proteins, and albumin), lipids (triglycerides, total cholesterol, HDL cholesterol, LDL cholesterol and free fatty acids). These parameters were determined using an Olympus analyzer with kits and controls supplied by Olympus or other suppliers (e.g. Wako for Free fatty acids).

Insulin measurement was performed using a mouse ultra-sensitive insulin Elisa kit (Mercodia, Uppsala, Sweden).

Necropsy

Organs were collected into 2 ml barcoded tubes (CORNING) and the barcodes were transferred into an Excel data sheet using a GRYPHON^tm^ reader. After CO_2_ euthanasia, each mouse was measured and weighed. Blood was collected by cardiac puncture and transferred into heparin tubes. Two people performed necropsies on each mouse to minimize the time of dissection (approximately 15 minutes); one person dissected the brain, kidneys, and heart and the other collected the rest of the organs. The brain was dissected as shown in the schematic below. The sample for tube 1A was fixed in 10 % formalin and samples for tubes 1B, 2, 3, and 4 were snap frozen. The two kidneys were decapsulated using fine forceps and weighed together. They were then cut transversally into five pieces; the two extremities were thrown away and the other three pieces were dissected to separate the cortex (outer layer, light brown) and medulla (inner layer, white). The cortexes and medullas of the two kidneys were pooled and snap frozen in two tubes. The heart was weighed then a transversal cut was made between the auricles and ventricles and the auricles were discarded. By positioning the heart on the apex to see the upper part, the right ventricular free wall was separated from the left ventricle and septum and these two samples were weighed and snap frozen. The entire liver (without gall bladder) was weighed, then the left lateral lobe and the rest of the liver were snap frozen separately for profiling and triglyceride measurements respectively. Subcutaneous, gonadal, and mesenteric fat were weighed and snap frozen and the quadriceps was snap frozen.

Triglycerides in liver

After lipid extraction according to the Folch method (chloroform/methanol), liver triglycerides were evaluated by the enzymatic color test using commercial reagents (Biomerieux, France).

eSNP Analysis for the Obsesity Cohort

The cohort was comprised of patients who underwent RXY gastric bypass surgery. Subcutaneous adipose and omental adipose tissues were collected from each patient at the time of surgery at Massachusetts General Hospital. Genomic DNA was extracted from liver tissues, and total RNA was extracted from liver, subcutaneous adipose, and omental adipose tissues. Each DNA sample was genotyped on the Illumina 650Y BeadChip array and each RNA sample was profiled on the custom 44K Agilent array. In total, 950 samples were successfully genotyped. IBS analysis was performed to identify related individuals. Eighteen parent-offspring, six sibling, and eight second degree relatives were identified, four of which were related trios. Twenty-eight individuals were removed to eliminate IBS in the dataset, leaving 922 samples for use in the analysis. Successful gene expression profiling results were collected from 707 liver samples, 916 omental adipose samples, and 870 subcutaneous adipose samples.

Demographic information including age, race, sex, height, type of surgery, and year of surgery were collected for each patient. All surgeries occurred between 2000 and 2007, with 59% of the surgeries performed laproscopically and the rest by open surgery. The independent contribution of the covariates age, race, sex, surgery year, surgery type, and BMI were analyzed. The distribution of *p*-values obtained from an ANOVA model for each covariate strongly suggested that it was necessary to correct for age, race, sex, and surgery year. By removing the contribution of surgery year the surgery type became unimportant, suggesting a strong correlation between these two covariates***.*** This is most likely because the surgery type was changed from open surgery to a laparoscopic approach during the course of sample collection. BMI was shown to have a normal distribution.

Four µg of total RNA from each treated sample was used to synthesize dsDNA through reverse transcription. cRNA was produced by *in vitro* transcription and labeled post-synthetically with Cy3 or Cy5. cRNA derived from cells treated with growth factor or AKT inhibitor (experimental sample) was hybridized against cRNA derived from an equal mix of all the samples (reference sample). Two hybridizations were performed with each cRNA sample. Pair probes on the microarrays were synthesized *in situ* using inkjet technology (Agilent Technologies, Palo Alto, CA). After hybridization, arrays were scanned and fluorescence intensities for each probe were recorded. Ratios of transcript abundance (experimental to control) were obtained following normalization and correction of the array intensity data. Gene expression data was analyzed using Rosetta Resolver gene expression analysis software (version 7.0, Rosetta Biosoftware, Seattle, WA) and MATLAB (The MathWorks, Natick, MA).

All expression traits were tested for association with each of the genotyped SNPs meeting the filtering criteria discussed above. The strongest putative *cis* eQTL for a given expression trait was defined as the SNP most strongly associated with the expression trait among all of the SNPs typed within 1 megabase (Mb) of the transcription start or stop of the corresponding structural gene. The association *p*-values were adjusted to control for testing of multiple SNPs and expression traits using an empirically determined false discovery rate (FDR) constrained to be < 10%. In the case of trans eQTL, all SNPs were tested for association to each of the expression traits.
